# Water-soluble allyl sulfones for dual site-specific labelling of proteins and cyclic peptides†
†Electronic supplementary information (ESI) available. See DOI: 10.1039/c6sc00005c


**DOI:** 10.1039/c6sc00005c

**Published:** 2016-01-29

**Authors:** Tao Wang, Andreas Riegger, Markus Lamla, Sebastian Wiese, Patrick Oeckl, Markus Otto, Yuzhou Wu, Stephan Fischer, Holger Barth, Seah Ling Kuan, Tanja Weil

**Affiliations:** a Institute of Organic Chemistry III , Ulm University , Albert-Einstein-Allee 11 , D-89081 Ulm , Germany . Email: Tanja.Weil@uni-ulm.de; b Core Unit Mass Spectrometry and Proteomics , University of Ulm Medical Center , D-89081 Ulm , Germany; c Department of Neurology , University of Ulm Medical Center , Oberer Eselsberg 45 , D-89081 Ulm , Germany; d Institute of Pharmacology and Toxicology , University of Ulm Medical Center , Albert-Einstein-Allee 11 , D-89081 Ulm , Germany

## Abstract

Water-soluble allyl sulfones provide convenient site-specific disulfide rebridging of native proteins and cyclic peptides. The site-selective functionalization of (a) the peptide hormone somatostatin, (b) the interchain disulfide of bovine insulin and (c) functionalization of the proteins GFP and lysozyme with allyl sulfones proceeds in aqueous solution. Allyl sulfones offer three functionalizable sites that react with thiol containing molecules in a step-wise fashion. Dual labeling of proteins and cyclic peptides is achieved *i.e.* the attachment of a chromophore and an affinity tag in a single reaction step, which is of great significance for the construction of precise multifunctional peptide and protein conjugates.

## Abstract 2

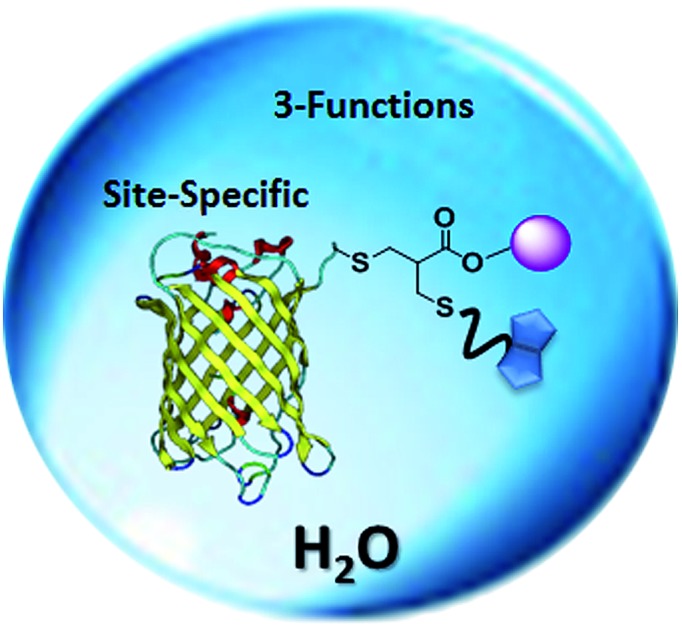
Allyl sulfones as efficient disulfide rebridging agents for site-specific protein modifications with up to two additional functionalities in water.

## Introduction

**Scheme 1:**
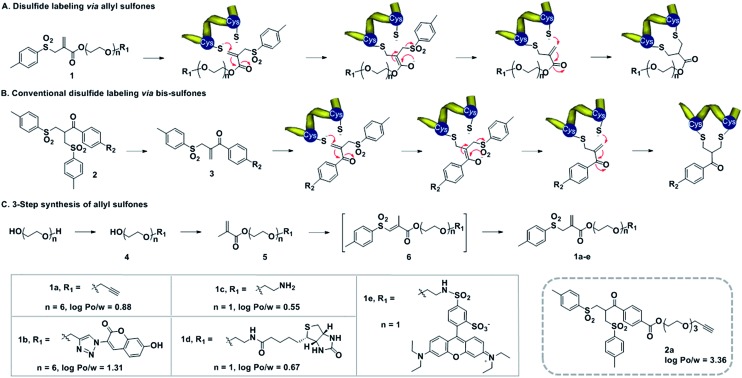
(A) Mechanism of disulfide rebridging by allyl sulfones **1**, involving two consecutive Michael additions. (B) Bis-sulfones **2** require first an activation to form the mono-sulfones instead. (C) Synthesis of allyl sulfones with different functionalities and the log *P*_o/w_ values predicted using ChemBiodraw 2013.

The spatially defined chemical modification of proteins represents a vibrant field of research, with great impact on various research areas such as the elucidation of protein functions,^1^ monitoring cellular processes,^2^ the development of new biocatalysts,^3^ the construction of biomaterials^4^ and the generation of novel therapeutics.^5^ For most of these applications, reproducible and well-defined protein conjugates need to be achieved with retained structural integrity and biological function. Despite great progress in this area, the modification of native proteins at a distinct location still represents a major challenge. Methods for site-selective protein modification can be roughly divided into two categories, with the first targeting a specific amino acid at the protein surface based on its abundance and accessibility. The relatively rare amino acid cysteine is a popular target for single-site modification.^6^ However, only very few proteins offer accessible, unpaired cysteine residues and consequently cysteine point mutations need to be introduced.^7^ N-terminal modification represents a powerful approach, which has certain limitations if the terminus is critical for function.^8^ The second method involves the incorporation of unnatural amino acid, allowing subsequent chemical modification with a high level of site-selectivity.^9^ However, tedious synthesis of aminoacylated tRNA can limit its general applicability.^10^ As a complementary strategy, disulfide rebridging represents a versatile technique facilitating the selective modification of accessible disulfides on proteins and peptides.^11^,^12^ This strategy involves a Michael acceptor system and two cysteines in close vicinity and it is particularly attractive for medicinal applications,^13^ since most therapeutically relevant proteins offer at least one disulfide bond close to the surface.^14^ Disulfide rebridging reagents such as bis-sulfones (Scheme 1B) and dibromomaleimides have been applied for the functionalization of peptides^13^,^15^,^16^ and Fab fragments^17^,^18^ that provide just one accessible disulfide bridge. Additionally, multiple disulfides of antibodies^18^,^19^ and therapeutic proteins^20^ have been functionalized previously. Bioconjugation reagents combining both the maleimide and bis-sulfone function offer efficient cross-conjugation of two different thiol-containing proteins, peptides or oligonucleotides.^21^ The bis-sulfide bioconjugates produced by disulfide rebridging reagents can be disintegrated under certain stimuli, which offers great potential in biomedical sensing, medical diagnosis and controlled drug release.^22^,^23^ However, until now, both low water-solubility and reactivity have limited the application of bis-sulfones and organic–aqueous co-solvents need to be added, which could lead to denaturation of the protein structure.^15^ In addition, disulfide functionalization with bis-sulfones usually yields the mono-functionalized protein and the attachment of multiple functionalities to a single disulfide bridge has not been achieved yet.^24^–^26^ Herein, we first introduce allyl sulfones as efficient disulfide rebridging agents providing improved reactivity without *in situ* activation, stability, high water-solubility and site-specificity for precise protein modifications with up to two additional functionalities. We demonstrate the broad applicability of our approach on the basis of the peptide hormone somatostatin (SST), bovine insulin as well as lysozyme with one, three and four disulfide bridges, respectively. Excellent site-selectivity and predictability allow the functionalization of the most solvent accessible disulfide at the protein surface by retaining functional activity. Noteworthy, site-specific disulfide modification of insulin and lysozyme is demonstrated for the first time. Allyl sulfones offer the combination of up to three different functionalities at a single site in a modular fashion. In this way, access to customized bioconjugates is granted by *e.g.* attaching a chromophore and a purification tag in a single step by simply adjusting the pH.

## Results and discussion

### Design of allyl sulfones for protein modification

Water-soluble allyl sulfones **1** were designed based on the essential features of disulfide rebridging agents, which include an effective leaving group (*e.g. p*-toluene sulfonyl group and halogen) and an electron-withdrawing keto-group conjugated to a double bond. Allyl sulfones **1** rebridge the most accessible disulfides of peptides and proteins after disulfide reduction *via* sequential addition–elimination reactions (Scheme 1A). First, the mild reduction of a solvent accessible disulfide releases two free thiols in close vicinity. Next, allyl sulfones **1** react by thiol addition of the double bond with subsequent elimination of the *p*-toluene sulfinic acid group to form a second Michael system. The second thiol group then undergoes the next Michael addition reaction under concomitant formation of the three-carbon bridge. Compared to the reported and widely used bis-sulfones **2a**, the allyl sulfones **1a–e** lack two hydrophobic benzene groups, which provide low *n*-octanol–water partition coefficients (log *P*_o/w_) indicating improved water-solubility (Scheme 1). They also offer more effective disulfide rebridging, since bis-sulfones **2** require *in situ* activation to form the reactive mono-sulfones **3** and this equilibrium step is usually not quantitative (Scheme 1B).

Allyl sulfones **1a–e** were readily obtained following a convenient three step reaction sequence (Scheme 1C). A short triethylene glycol or hexaethylene glycol chain was mono-substituted with the desired functionality and then condensed with methacrylchloride to form the corresponding methacrylate **5**. Allyl sulfones **1a–e** were synthesized *via* a tandem iodosulfonylation–dehydroiodination reaction.^27^ This one-pot reaction first generated the intermediate vinyl sulfones **6**, which isomerized to the thermodynamically more stable allyl sulfones **1a–e** under reflux and basic conditions.^27^ Ethynyl- and amino-allyl sulfones **1a** and **1c** are attractive building blocks for generating further rebridging reagents with the desired functionalities by Cu(i) catalyzed cycloaddition and condensation reactions, respectively.

### Site-directed functionalization of somatostatin, bovine insulin and lysozyme

**Fig. 1:**
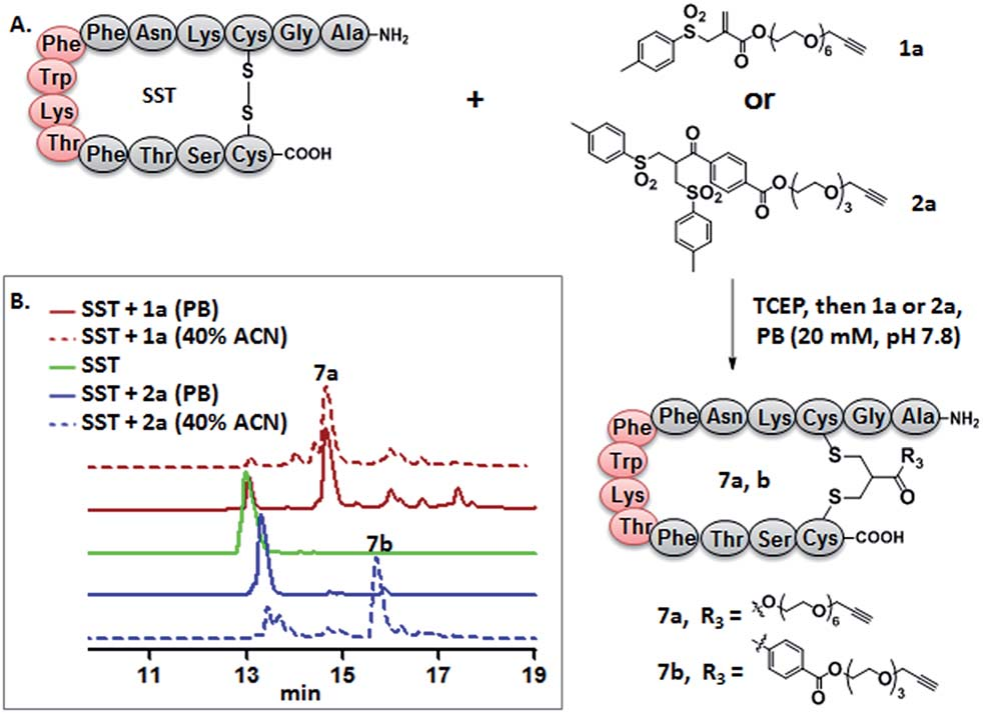
(A) Disulfide rebridging of SST by allyl sulfone **1a** proceeds in buffer in comparison to bis-sulfone **2a** requiring 40% ACN. (B) The HPLC profile of disulfide rebridging of SST by **1a** or **2a** in phosphate buffer with or without 40% ACN.

Somatostatin, bovine insulin and lysozyme were selected for site-directed modifications with allyl sulfones, which contain one, three and four disulfide bridges, respectively. Disulfide rebridging of the peptide hormone somatostatin (SST) was accomplished by allyl sulfone **1a** and bis-sulfone **2a** in aqueous and organic/aqueous solutions, respectively (Fig. 1). Disulfide rebridging using bis-sulfone **2a** required at least 40% acetonitrile (ACN), whereas the reaction with allyl sulfone **1a** proceeded in aqueous media (Fig. 1B). The corresponding products **7a** and **7b** were purified by HPLC in 49% and 41% yield, respectively, and characterized *via* HR-MALDI-TOF MS (Fig. S23†). The ester linkage of **7a** showed high stability to hydrolysis at physiologically pH (pH 6–8, Fig. S2†) and even in 10% FCS solution (Fig. S3†) up to 48 h. The functionality of the introduced alkyne of somatostatin **7a** was demonstrated in a copper catalyzed cycloaddition reaction with azido coumarin (Fig. S1 and S24†).

**Fig. 2:**
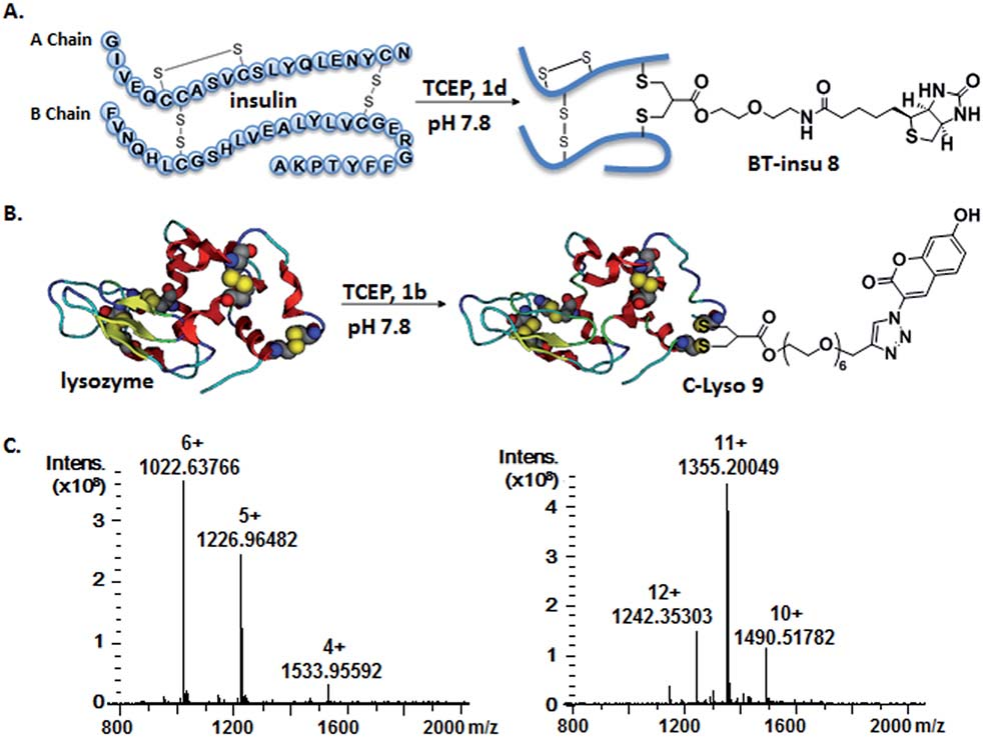
Site-specific modification of bovine insulin (A) and lysozyme (B) *via* disulfide rebridging. (C) HR-ESI mass spectrum of BT–insu **8** (left) and C–Lyso **9** (right).

**Fig. 3:**
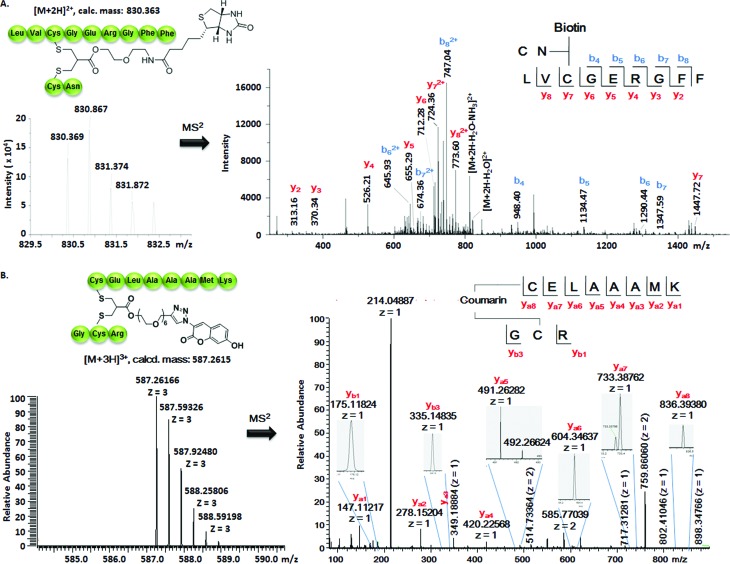
(A) Full interpretation of the MS^2^ spectrum of the peptide fragments resulted from the chymotrypsin digestion of BT–insu **8**. (B) Full interpretation of the MS^2^ spectrum of the peptide fragments resulted from the trypsin digestion of C–Lyso **9**. Detailed experimental conditions and the origin of the peptide fragments are given in the ESI.†

Insulin is a polypeptide hormone excreted by the pancreas, which plays a crucial role in carbohydrate and fat metabolism and it offers three disulfides.^28^ Very recently, Loh *et al.* have demonstrated selective cysteine modification on reduced bovine insulin using allenamides in ammonium carbonate buffer containing 33% THF, resulting in two separate, fully modified chains A and B.^29^ Herein, allyl sulfone **1d** selectively “reannealed” the interchain disulfide bond of bovine insulin in aqueous buffer as shown in Fig. 2. Bovine insulin was treated with 1.2 equiv. of the mild reduction reagent tris(2-carboxyethyl)phosphine (TCEP) and 2 equiv. of biotin–allyl sulfone **1d** sequentially at pH 7.8 and the resulting reaction mixture was incubated at RT for 24 h (Fig. 2A). The product biotin–insulin (BT–insu, **8**) was purified *via* HPLC in 28% yield. 40% of native insulin was successfully recovered during HPLC purification and could be recycled for subsequent functionalization of insulin. The isolated BT–insulin **8** exhibited signals with multiple charges at 1022.63766 [M + 6H]^6+^, 1226.96482 [M + 5H]^5+^, 1533.95592 [M + 4H]^4+^ and the average mass was determined as 6131.96416 after deconvolution (Fig. 2C, left). The average mass (Δ*m*/*z* 399 relative to bovine insulin) matched the calculated M.W. of 6132.7. After in-solution digestion of BT–insulin **8** by chymotrypsin, peptide fragments were obtained and analyzed by LC-MS^2^ (Fig. 3A). The modification site was determined as the C20 (chain A)–C19 (chain B), which is consistent with the higher surface accessibility calculation implemented in the software package *Molecular Operating Environment* (MOE, Fig. S4†).^30^ Due to the improved solubility and reactivity of allyl sulfones, more than one disulfide modification could be accomplished if desired. By applying the reducing agent and **1a** in excess to bovine insulin, the attachment of up to three bioconjugation reagents to different disulfides has been detected suggesting that even a step-by-step rebridging could be achieved, which paves the way to dual or even higher modified insulin derivatives (Fig. S25†).

We further tested allyl sulfone **1b** for the site-specific modification of the enzyme lysozyme (from hen egg white). Lysozyme (1,4-β-*N*-acetylmuramidase) hydrolyzes the β (l–4) glycosidic bonds, and induces bacterial cell death through lysis of the cell walls. It has received great interest for various applications in medicine, cosmetics and food industry due to its anti-bactericidal activity. Moreover, it is a disulfide rich protein, contains four disulfide bonds and the disulfide C6–C127 is predicted to be most solvent accessible based on the calculation of MOE (Fig. S5†). Lysozyme was treated with 1.05 equiv. of TCEP followed by 2 equiv. of allyl sulfone **1b** and the resulting reaction mixture was incubated at RT for 24 h (Fig. 2B). The product coumarin–lysozyme **9** (C–Lyso) was isolated in 19% yield by FPLC (ÄKTA from GE) using a Hi Trap phenyl HP column (1 mL, from GE) based on the hydrophobicity. Noteworthy, 30% of native lysozyme was recovered during the FPLC purification, which was again recycled for further functionalization. Protein modification often proceeds in low yields and without further purification. Herein, the yield of modified lysozyme was assessed after purification. In comparison, the only unpaired and accessible cysteine residues of HSA and BSA,^31^ have been functionalized with 30% labeling efficiency.

The HR ESI-MS spectrum confirms mono-modification of lysozyme with the corresponding multiply-charged signals at *m*/*z* 1242.35303 [M + 12H]^12+^, 1355.20049 [M + 11H]^11+^, 1490.51782 [M + 10H]^10+^ (Fig. 2C, right). The average mass of C–Lyso was determined as 14 897.33283 [M + H]^+^ with a difference of 409.19 relative to native lysozyme after deconvolution, matching the calculated M.W. of 14 896.9. The modification site was determined by trypsin digestion of C–Lyso **9**, followed by the detection of the fragments in HR-MALDI-MS. The fragment with *m*/*z* 1759.77223 [M + H]^+^ corresponded well to the calculated M.W. of 1759.76996, confirming that the disulfide C6–C127 was rebridged with the coumarin functionality (Fig. S6†). HR-ESI-MS also revealed a decarbonylation fragment with mass signals at 1731.76513 (calcd mass: 1731.77504), due to decarbonylation from the pyrone ring of the coumarin to form a benzofuran ring, as reported before.^32^ The coumarin modified peptide fragment was then sequenced by LC-MS^2^, which again demonstrated modification of disulfide C6–C127 (Fig. 3B). C–Lyso **9** has similar circular dichroism (CD) spectra as native lysozyme, indicating that the secondary and the tertiary structures of lysozyme remained unchanged after modification (Fig. S7†). Finally, the catalytic activities of C–Lyso **9** and native lysozyme were recorded applying a lysozyme activity kit (Sigma Aldrich, Cat. no. LY0100) according to the manufacturer's instructions. The catalytic activities of C–Lyso and lysozyme have been determined as 367.7 ± 16.5 units per nmol and 412.7 ± 21.0 units per nmol, respectively, demonstrating that lysozyme's functional activity was retained after modification (Fig. S8, Table 1†). These results underline that disulfide rebridging of proteins *via* allyl sulfones **1** proceeds with high selectivity, efficiency, under mild aqueous conditions and without perturbation of the tertiary structure and function of lysozyme.

### Allyl sulfones as reactive sites facilitating protein multifunctionalization

**Fig. 4:**
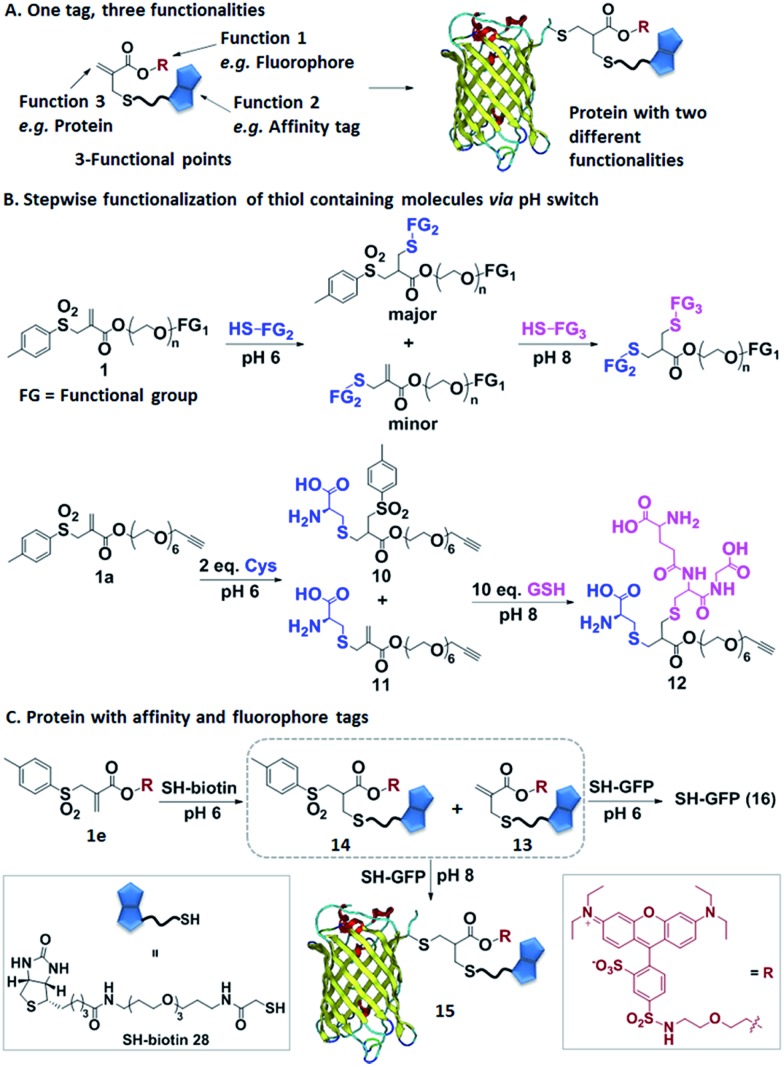
(A) Allyl sulfones offer dual labeling of proteins. (B) Illustration of the step-wise coupling of two thiol containing molecules by simply adjusting the pH; depiction of the model reaction. (C) Allyl sulfone **1e** allows step-wise conjugation of SH-biotin and SH-GFP by increasing the pH from 6 to 8.

In the next step, we have explored allyl sulfones **1** as versatile building blocks for convenient preparation of more complex bioconjugates containing up to three different functionalities (Fig. 4A). Bis-sulfones react with thiols at pH 8 by *in situ*-elimination of *p*-toluene sulfinic acid yielding a monosulfone.^21^ In contrast, allyl sulfones undergo a Michael reaction with thiols already at pH 6 and at pH 8 in a sequential fashion (Fig. 4B), which was monitored by LC-MS. First, allyl sulfone **1a** reacted with 2 equiv. cysteine at pH 6 forming the mono-cysteine adduct **10**. This reaction was already completed after only 15 min reaction time (Fig. S9†). Only minor traces of the *p*-toluene elimination product **11** and no bis-cysteine adduct **17** were detected within 1 h. However, at pH 8 and after 1 h incubation, the elimination product **11** and the bis-cysteine adduct **17** were formed as major products (Fig. S9†). In the second step, the mono-cysteine adduct **10** was further reacted with 10 equiv. of glutathione (GSH) at pH 6 and pH 8. At pH 8, the reaction with GSH yielded product **12** almost quantitatively, while at pH 6, no reaction occurred even after 24 h of incubation (Fig. S10†). Therefore, optimal conjugation conditions require reaction with the first thiol containing molecule at pH 6 for about 1 h reaction time followed by the addition of the second thiol containing molecule at pH 8 and incubation for 24 h.

Allyl sulfone **1e** has been used to derivatize the single cysteine at the recombinant green fluorescent protein (SH-GFP) with a single biotin tag and a fluorescent probe (Fig. 4C). Such modifications are attractive since they combine purification *via* affinity chromatography (*e.g.* avidin beads) and a fluorescence tag for detection. Compound **1e** was treated with 1.5 equiv. of SH-biotin (**28**) at pH 6 for 1 h and the product was purified by column chromatography. The resultant products were characterized by LC-MS. Elimination of *p*-toluene sulfinic acid already occurred during column chromatography, resulting in the detection of the major product **13** (*m*/*z* = 617 [M + 2H]^2+^) and very minor quantities of product **14** (*m*/*z* = 695 [M + 2H]^2+^) (Fig. S11†). Both **13** and **14** (20 equiv.) were further reacted with SH-GFP at pH 8 overnight at RT. The modified GFP **15** was purified by size exclusion chromatography using a Sepharose G-25 matrix and Milli-Q water as eluting solvent in 90% yield. Successful conjugation was demonstrated by gel electrophoresis, optical spectra, and the detection of biotin *via* western blotting (Fig. S12†). Gel electrophoresis analysis proved the formation of conjugate **15** due to the intensive fluorescent band at 30–31 kDa corresponding well to the M.W. of 30 511 g mol^–1^ (Fig. S12A†). Excitation of the conjugate **15** at 492 nm revealed a decrease of GFP fluorescence at 512 nm and an increase of the lissamine rhodamine B (Rho B) emission at 594 nm, indicating energy transfer from GFP to Rho B (Fig. S12C†) due to the presence of both chromophores within one molecule. Based on the absorption spectra and extinction coefficients, the labeling efficiency of 55% of the Rho B on SH-GFP was calculated (ESI†). The labeling of biotin on GFP was verified by western blotting (Fig. S12B†). To confirm that the biotin group also has similar labeling efficiency as Rho B, the conjugate **15** was incubated with streptavidin agarose and 91% of the conjugate was immobilized on the streptavidin agarose (ESI†). In contrast, no conjugation occurred at pH 6 according to SDS-PAGE, optical spectra and western blotting (Fig. S12†).

## Conclusions

The convenient three-step synthesis of water-soluble allyl sulfones yields versatile and efficient reagents for the spatially defined modification of bioactive cyclic peptides and proteins under mild, aqueous conditions preserving tertiary structure and function. The reaction could even be conducted at low temperature, which is particularly important for many temperature-sensitive proteins such as antibodies. Compared to known bis-sulfones, disulfide rebridging using allyl sulfones proceeds more efficiently since *in situ* activation is not required anymore. The improved solubility and reactivity of allyl sulfones facilitated the site-specific disulfide functionalization of native proteins insulin and lysozyme for the first time. Moreover, allyl sulfones offer the unique opportunity to conjugate two additional thiol containing molecules to the thus modified protein in a step-wise fashion by simply increasing the pH from 6 to 8, which is attractive for imparting additional functions *e.g.* fluorescence and purification tags. As proof of concept, GFP was equipped with a single Rho B chromophore and a biotin affinity tag at a single site. We believe that our results are of great significance for the construction of precise multifunctional peptide and protein conjugates, which is of emerging importance to achieve detectable protein therapeutics with *e.g.* improved pharmacokinetic parameters.

## Supplementary Material

Supplementary information
