# Supplementary material for: Water-soluble allyl sulfones for dual site-specific labelling of proteins and cyclic peptides
Source: Chem Sci. 2016 Jan 29;7(5):3234–9. doi: 10.1039/c6sc00005c (PMC6006486; doi:10.1039/c6sc00005c)
Supplement: Supplementary file 1 [file SC-007-C6SC00005C-s001.pdf]

Electronic Supplementary Material (ESI) for Chemical Science.
This journal is © The Royal Society of Chemistry 2016

Supporting Information

Tao Wang,[‡] Andreas Riegger,[‡] Markus Lamla, Sebastian Wiese, Patrick Oeckl, Markus Otto,Yuzhou Wu, Stephan Fischer, Holger Barth, Seah Ling Kuan and Tanja Weil*

Content

1. **General experimental.**
2. **Synthesis of disulfide rebridging reagents.**
   (1) **Synthesis of allyl sulfone with alkyne and coumarin functionalities.**
   (2) **Synthesis of allyl sulfone with amine, biotin and rhodamine functionalities.**
3. **Disulfide rebridging of somatostatin.**
   (1) **Synthesis and purification 7a, Post functionalization of 7a via CuAAc.**
   (2) **Stability tests of 7a in solutions with different pH and in 10% FCS.**
4. **Disulfide rebridging of bovine insulin.**
5. **Disulfide rebridging of lysozyme (chicken egg white).**
6. **Circular dichroism (CD) of C-lyso 9 and lysozyme.**
7. **Lysozyme activity assay.**
8. **Allyl sulfones 1 as three functional building block for step-wise conjugation of thiol containing molecules by adjusting pH.**
   (1) **Synthesis of the reagents.**
   (2) **LC-MS study for the model reaction.**
   (3) **Expression of GFP containing a single cysteine mutation.**
   (4) **Allyl sulfone 1e for step-wise conjugation of SH-biotin and SH-GFP by increasing the pH.**
9. **(HR)-MS spectra.**

1. General experimental.

Unless otherwise noted, all operations were performed without taking precautions to exclude air and moisture. All solvents and reagents were purchased from commercial sources and were used without further purification. In some cases, they have been distilled and dried before use. Reaction progress was monitored by thin layer chromatography (TLC) using Merck 60 F_{254} pre-coated silica gel plates illuminating under UV 254 nm or using appropriate stains (Ninhydrine, $KMnO_4$). Flash column chromatography was carried out using Merck silica gel 60 mesh. NMR spectra were measured on Bruker DRX 400 MHz or 500 MHz NMR spectrometer and the chemical shifts (δ) were referenced to residual solvent shifts in the respective deutero solvents. Chemical shifts are reported as parts per million referenced with respect the residual solvent peak. Chemical ionization mass spectra (CI-MS) were obtained

on a Finnigan MAT, SSQ-7000. MALDI-TOF-MS spectra were acquired on a Bruker Reflex III. HR-MALDI-MS and HR-ESI-MS were recorded on a Solarix (Bruker) FTICR-MS. LC-MS analysis was performed on a Shimadzu LC-MS 2020 equipped with an electrospray ionisation source and a SPD-20A UV-Vis detector (Shimadzu, Duisburg, Germany). The absorbance and emission were measured on Microplate Readers (Tecan Infinite®M1000 PRO). The nitrocellulose membrane is obtained from GE Healthcare Life Science, Fairfield, Connecticut, USA. Enhanced chemiluminescence (ECL) system was purchased from Millipore (Schwalbach, Germany).

2. Synthesis of disulfide rebridging reagents.

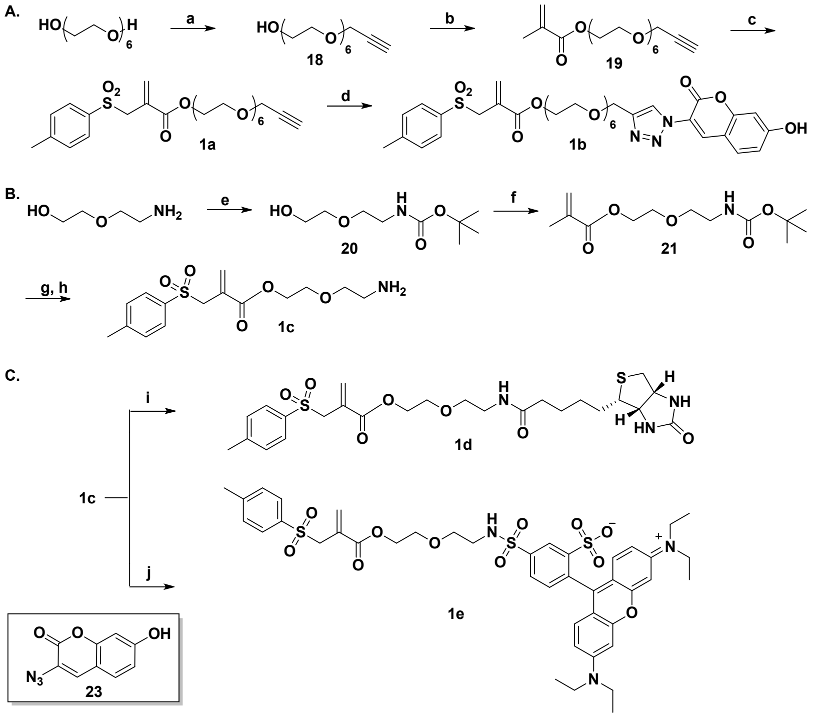

Scheme S1. The synthetic scheme of allyl sulfones with different functionalities. a. NaH, propargyl bromide, dry THF, 53%; b. NEt_3, methacrylchloride, dry DCM, 70%; c. 1.sodium *p*-toluenesulfinate, I_2, DCM, 3d, 2. NEt_3, DCM, overnight, 3. NEt_3, EA, 95℃, overnight, 50%; d. **23**, $CuSO_4$, Na ascorbate, THF/H_2O 1:1, overnight, 76%; e. NEt_3, Boc, DCM, 90%; f. NEt_3, methacrylchloride, dry DCM, 89%; g. 1.sodium *p*-toluenesulfinate, I_2, DCM, 3d, 2. NEt_3, DCM, overnight, 3. NEt_3, EA, 95℃, overnight, 81%; h. TFA, DCM, 98%; i. biotin, NEt_3, BOP, dry DMF, 77%; j. lissamine rhodamine b sulfonyl chloride, DIEA, dry DMF, 2d, 36%.

(1) Synthesis of allyl sulfone with alkyne and coumarin functionalities.

Synthesis of propargyl-hexaethylene glycol (18)

Under argon atmosphere, a stirred solution of hexaethylene glycol (5 g, 17.7 mmol, 2 equiv.) in dry THF was cooled to 0°C using an ice bath. NaH (460 mg, 11.6 mmol, 1.3 equiv, 60% suspension in petrol ether) was added in small portions. The reaction mixture was stirred for

30 min at 0°C, before 787 µL (8.85 mmol) of propargylbromide (80% in toluene) was added dropwise. After stirring the dispersion overnight, the THF was removed in vacuum and the mixture was purified by column chromatography with 4% MeOH in $CHCl_3$ to afford 1.49 g of the product **18** as a yellow liquid in 53% yield. **^{1}H-NMR** (400 MHz, $CDCl_3$): δ = 2.40 (t, 1H, *J* = 2.4 Hz), 2.96 (s, br, 1H), 3.50 – 3.66 (m, 24 H), 4.13 (d, 2H, *J* = 2.4 Hz) ppm. **^{13}C-NMR** (100 MHz, $CDCl_3$): δ = 58.31, 61.56, 69.00, 70.23, 70.31, 70.45, 70.47, 70.49, 70.52, 72.50, 74.60, 79.60 ppm. **HR ESI-FTICR-MS:** m/z = 343.17244 $[M+Na]^+$ (calcd. exact mass: 343.17272 $[M+Na]^+$, formula: $C_{15}H_{28}O_7$).

Synthesis of methacryl-propargyl-hexaethylene glycol (19)

In a 100 mL schlenk flask, propargyl-hexaethylene glycol **18** (1.45 g, 4.5 mmol) and NEt_3 (942 µL, 6.78 mmol, 1.5 equiv.) were dissolved in 40 mL of dry DCM and cooled to 0°C. Then methacrylchloride (656 µL, 6.78 mmol, 1.5 equiv) was added dropwise to the solution. After stirring overnight, the reaction mixture was filtered and the solvent was removed. The mixture was purified by column chromatography with EA/ Hexane (2:1) to afford 1.22 g of the ester **19** as colorless oil in 70% yield. **^{1}H-NMR** (400 MHz, $CDCl_3$): δ = 1.93 (dd, 3H, *J* = 1.0 Hz, *J* = 1.4 Hz), 2.42 (t, 1H,*J* = 2.4 Hz), 3.60 - 3.70 (m, 20H), 3.71 – 3.75 (m, 2H), 4.19 (d, 2H, *J* = 2.4 Hz), 4.26 – 4.31 (m, 2H), 5.56 (m, 1H), 6.12 (m, 1H) ppm. **^{13}C-NMR** (100 MHz, $CDCl_3$): δ = 18.41, 58.46, 63.96, 69.16, 69.20, 70.47, 70.57 – 70.75 (m, 8C), 74.64, 79.71, 125.84, 136.20, 167.43 ppm. **HR ESI-FTICR-MS:** m/z = 411.19881 $[M+Na]^+$, 427.17319 $[M+K]^+$ (calcd. exact mass: 411.19894 $[M+Na]^+$, 427.17288 $[M+K]^+$, formula: $C_{19}H_{32}O_8$).

Synthesis of 3,6,9,12,15,18-hexaoxahenicos-20-yn-1-yl 2-(tosylmethyl)acrylate (1a)

Methacryl-propargyl-hexaethylene glycol **19** (500 mg, 1.29 mmol) was dissolved in 12 mL of DCM. Sodium *p*-toluenesulfinate (344 mg, 1.93 mmol, 1.5 equiv.) and I_2 (490 mg, 1.93 mmol, 1.5 equiv.) were added sequentially. The reaction suspension was stirred for three days at room temperature before NEt_3 (537 µL, 3.86 mmol, 3 equiv.) was injected. After stirring overnight, the organic layer was washed with HCl, $NaHCO_3$, $Na_2S_2O_3$ and Brine solution. The organic layer was dried over $MgSO_4$ and the solvent was evaporated. The residue was dissolved in 15 mL of ethylacetate and NEt_3 (537 µL, 3.86 mmol, 3 equiv.) was added slowly at 0℃. The reaction mixture was warmed up to RT and refluxed overnight at 95°C. The solvent was removed and the crude product was purified by column chromatography with EA to afford 351 mg of the ethynyl-allyl sulfone **1a** as yellowish oil in 50% yield. **^{1}H-NMR** (400 MHz, $CDCl_3$): δ = 2.44 (s, 3H), 3.60 - 3.72 (m, 23H), 4.20 (d, 2H, *J* = 2.4), 4.10 - 4.16 (m, 4H), 5.89 (s, 1H), 6.52 (s, 1H), 7.33 (d, 2H, *J* = 8.1 Hz), 7.73 (d, 2H, *J* = 8.3 Hz) ppm. **^{13}C-NMR** (100 MHz, $CDCl_3$): δ = 21.79, 57.62, 58.48, 64.60, 68.86, 69.17, 70.48, 70.63 - 70.70 (m, 8C), 74.66, 79.74, 128.84, 128.99, 129.78, 133.71, 135.46, 144.96, 164.90 ppm. **LC-MS (ESI)**: m/z = 543 $[M+H]^+$, 560 $[M+H_2O]^+$ (calcd. mass: 542.22, formula: $C_{26}H_{38}O_{10}S$). **HR MALDI-FTICR-**

MS: m/z = 565.20710 $[M+Na]^+$, 581.18106 $[M+K]^+$ (calcd. exact mass: 565.20779 $[M+Na]^+$, 581.18173 $[M+K]^+$, formula: $C_{26}H_{38}O_{10}S$).

Synthesis of 3-azido-7-hydroxy-2H-chromen-2-one (23)[1]

A stirred mixture of 2,4-dihydroxybenzaldehyde (2 g, 14.5 mmol), *N*-acetylglycine (1.7 g, 15.5 mmol) and anhydrous sodium acetate (3.56 g) in 70 mL of acetic anhydrate was refluxed for four hours. The resulting mixture was poured onto ice to give a yellow precipitation, which was washed with ice water. Then the residue was refluxed in conc. HCl/ EtOH (2:1) for one hour. Thereafter, ice water was used to dilute the reaction mixture before it was cooled to 0°C. Under stirring, $NaNO_2$ (2.00 g, 29 mmol, 2 equiv.) was added and the reaction mixture was stirred for another 15 min, followed by adding NaN_3 (2.80 g, 43.5 mmol, 3 equiv.) in portions. After 20 min of stirring, the resulting precipitate was filtered off and washed with ice water to afford 450 mg of the azido cumarin **23** as brown solid in 17% yield. **^{1}H-NMR** (400 MHz, $CDCl_3$): δ = 6.76 (d, 1H, *J* = 2.2 Hz), 6.81 (dd, 1H, *J* = 8.5 Hz, *J* = 2.3 Hz), 7.48 (d, 1H, *J* = 8.5 Hz), 7.60 (s, 1H) ppm. **^{13}C-NMR** (100 MHz, $CDCl_3$): δ = 102.07, 111.37, 113.82, 121.15, 127.87, 129.13, 152.79, 157.36, 160.33 ppm. **LC-MS (ESI):** m/z = 202 $[M-H]^-$ (calcd. mass: 203.03, formula $C_9H_5N_3O_3$).

Synthesis of 1-(1-(7-hydroxy-2-oxo-2H-chromen-3-yl)-1H-1,2,3-triazol-4-yl)-2,5,8,11, 14,17-hexaoxanonadecan-19-yl 2-(tosylmethyl)acrylate (1b)

Azido coumarin **23** (31.4 mg, 0.16 mmol) and ethynyl-allyl sulfone **4a** (100 mg, 0.18 mmol, 1.1 equiv.) were dissolved in 500 µL of THF and the mixture was further diluted with 500 µL of H_2O. Thereafter, sodium ascorbate (33 mg, 0.16 mmol, 1 equiv.) and $CuSO_4$ (5.1 mg, 0.032 mmol, 0.2 equiv.) were added sequentially. The reaction mixture was stirred overnight at RT. The suspension was filtered and the solvent was evaporated in vacuum. The mixture was purified by column chromatography with 2% MeOH in DCM to afford 90 mg of the coumarin allyl sulfone **1b** as yellow oil in 76% yield. **^{1}H-NMR** (400 MHz, $CDCl_3$): δ = 2.42 (s, 3H), 3.55 – 3.69 (m, 18H), 3.70- 3.80 (m, 4H), 4.10 – 4.15 (m, 4H), 4.72 (s, 2H), 5.89 (s, 1H), 6.50 (s, 1H), 6.80 – 6.88 (m, 2H), 7.32 (d, 2H, *J* = 8.3 Hz), 7.39 (d, 1H, *J* = 8.5 Hz), 7.71 (d, 2H, *J* = 8.3 Hz), 8.34 (s, 1H), 8.47 (s, 1H) ppm. **^{13}C-NMR** (100 MHz, $CDCl_3$): δ = 21.79, 57.68, 64.50, 64.62, 68.89, 69.94, 70.4 - 70.8 (9 C), 103.18, 110.62, 115.13, 119.32, 124.01, 128.85, 128.94, 129.86, 130.23, 133.86, 134.01, 135.46, 144.68, 145.08, 154.68, 156.20, 162.30, 164.98 ppm. **LC-MS (ESI)**: m/z = 746 $[M+H]^+$ (calcd. mass: 745.25, formula: $C_{35}H_{43}N_3O_{13}S$). **HR ESI-FTICR-MS:** m/z = 768.24050 $[M+Na]^+$, 784.21555 $[M+K]^+$ (calcd. exact mass: 768.24088 $[M+Na]^+$, 784.21482 $[M+K]^+$, formula: $C_{35}H_{43}N_3O_{13}S$).

(2) Synthesis of allyl sulfone with amine, biotin and rodamine functionalities

Synthesis of *tert*-butyl (2-(2-hydroxyethoxy)ethyl)carbamate (20)

In a 100 mL round bottom flask, 2-(2-aminoethoxy)ethanol (0.95 mL, 9.51 mmol) was dissolved in 30 mL of DCM and NEt_3 (1.59 mL, 11.41 mmol, 1.2 equiv.) was added. Thereafter, 2.44 mL of di-*tert*-butyl dicarbonate was dropped slowly to the solution. The reaction mixture was stirred overnight and the solvent was removed in vacuum. The residue was purified by column chromatography with EA/ Hexane (2:1) to afford 1.76 g of the product **20** as colorless oil in 90% yield. **^{1}H-NMR** (400 MHz, $CDCl_3$): δ = 1.40 (s, 9H), 3.05 (s, br, 1H), 3.23 – 3.33 (m, 2H), 3.47 – 3.55 (m, 4H), 3.65 – 3.72 (m, 2H), 5.25 (s, br, 1H) ppm. **^{13}C-NMR** (100 MHz, $CDCl_3$): δ = 28.45, 40.39, 61.63, 70.36, 72.33, 79.38, 156.27 ppm. **HR ESI-FTICR-MS:** m/z = 228.12049 $[M+Na]^+$, 244.09445 $[M+K]^+$, 433.25173 $[2M+Na]^+$ (calcd. exact mass: 228.12063 $[M+Na]^+$, 244.09457 $[M+K]^+$, 433.25204 $[2M+Na]^+$, formula: $C_9H_{19}NO_4$).

Synthesis of 2-(2-((tert-butoxycarbonyl)amino)ethoxy)ethyl methacrylate (21)

In a 250 mL schlenk flask, *tert*-butyl (2-(2-hydroxyethoxy)ethyl)carbamate **20** (3.58 g, 11.18 mmol) and NEt_3 (2.33 mL,16.78 mmol, 1.5 equiv.) were dissolved in 60 mL of dry DCM and cooled to 0°C. Then methacrylchloride (1.62 mL, 16.78 mmol, 1.5 equiv.) was added dropwise to the solution. After stirring overnight at RT, the reaction mixture was filtered and the solvent was removed. The residue was purified by column chromatography with EA / Hexane(1:4) to afford 2.72 g of the ester **21** as colorless oil in 89% yield. **^{1}H-NMR**(400 MHz, $CDCl_3$): δ = 1.43 (s, 9H), 1.93 - 1.96 (m, 3H), 3.25 – 3.35 (m ,2H), 3.54 (t, 2H, *J* = 5.1 Hz), 3.69 (t, 2H, *J* = 4.9 Hz), 4.29 (t, 2H, *J* = 4.7 Hz), 4.91 (s,br, 1H), 5.56 - 5.59 (m,1H), 6.11 - 6.14 (m, 1H) ppm. **^{13}C-NMR** (100 MHz, $CDCl_3$): δ = 18.47, 28.51 (3C), 40.42, 63.80, 68.98, 70.24, 79.40, 126.02, 136.20, 156.07, 167.49 ppm. **HR ESI-FTICR-MS:** m/z = 296.14668 $[M+Na]^+$, 312.12068 $[M+K]^+$, 569.30402 $[2M+Na]^+$, 585.27835 $[2M+K]^+$ (calcd. exact mass: 296.14684 $[M+Na]^+$, 312.12078 $[M+K]^+$, 569.30447 $[2M+Na]^+$, 585.27840 $[2M+K]^+$, formula: $C_{13}H_{23}NO_5$).

Synthesis of the 2-(2-aminoethoxy)ethyl 2-(tosylmethyl)acrylate (1c)

Step g: Compound **21** (1.00 g, 3.6 mmol) was dissolved in 36 mL of DCM. Sodium *p*-toluenesulfinate (978 mg, 5.4 mmol, 1.5 equiv.) and I_2 (1.39 g, 5.4 mmol, 1.5 equiv.) were added sequentially. The reaction suspension was stirred for three days at room temperature before 1.53 mL NEt_3 was injected slowly at 0℃. After stirring overnight, the organic layer was washed with H_2O, $NaHCO_3$, $Na_2S_2O_3$ and Brine solution. The organic layer was dried over anhydrous $MgSO_4$ and the solvent was evaporated. The residue was dissolved in 15 mL of ethylacetate and 1.53 mL of NEt_3 was added. The mixture was then refluxed overnight at 95 °C and the solvent was removed under vacuum. The residue was purified by column chromatography with EA/ Hexane(1:1.5) to afford 1.25 g of the product **22** as yellow oil in 81% yield. **^{1}H-NMR** (400 MHz, $CDCl_3$): δ = 1.42 (s, 9H), 2.43 (s, 3H), 3.25 – 3.35 (m, 2H), 3.51 (t, 2H, *J* = 5.2 Hz), 3.58 - 3.62 (m, 2H), 4.12 - 4.18 (m, 4H), 4.96 (s, br, 1H), 5.85 (s, 1H), 6.50 (s, 1H), 7.32 (d, 2H, *J* = 8.2 Hz), 7.73 (d, 2H, *J* = 8.3 Hz) ppm. **^{13}C-NMR** (100 MHz,

$CDCl_3$): δ = 21.78, 28.50, 40.40, 57.74, 64.45, 68.65, 70.27, 79.40, 128.85, 129.02, 129.81, 133.71, 135.50, 145.02, 156.08, 164.96 ppm. The oil was used for the next step without further characterization. **Step h:** In a 50 mL round bottom flask, compound **22** (1.00 g, 2.3 mmol) was dissolved in 20 mL of DCM and TFA (3.86 mL, 50.5 mmol, 20 equiv.) was added. The resulting mixture was stirred overnight at RT. The solvent and TFA was removed in vacuum to obtain 750 mg of the amine-allyl sulfone **1c** as yellow-brown oil in 98% yield. **^{1}H-NMR** (400 MHz, $CDCl_3$): δ = 2.43 (s, 3H), 3.16 - 3.28 (m, 2H), 3.68 – 3.78 (m, 4H), 4.14 (s, 2H), 4.30 - 4.37 (m, 2H), 5.55 (s, 1H), 6.44 (s, 1H), 7.33 (d, 2H, *J* = 8.0 Hz), 7.68 (d, 2H, *J* = 8.0 Hz) ppm. **^{13}C-NMR** (100 MHz, $CDCl_3$): δ = 21.78, 39.92, 58.09, 64.05, 66.30, 69.11, 128.66, 128.84, 134.09, 134.59, 145.54, 165.33 ppm. **LC-MS (ESI):** m/z = 328 $[M+H]^+$ (calcd. mass: 327.11, formula: $C_{15}H_{21}NO_5S$). **HR ESI-FTICR-MS:** m/z = 328.12132 $[M+H]^+$, 655.23515 $[2M+H]^+$ (calcd. exact mass: 328.12132 $[M+H]^+$, 655.23536 $[2M+H]^+$, formula: $C_{15}H_{21}NO_5S$).

Synthesis of 2-(2-(5-((3aS,4S,6aR)-2-oxohexahydro-1H-thieno[3,4-d]imidazol-4-yl) pentanamido)ethoxy)ethyl 2-(tosylmethyl)acrylate (1d)

Under argon atmosphere, a stirred solution of biotin (77 mg, 0.31 mmol) and triethylamine (87 µL, 0.62 mmol, 2 equiv.) in 3 mL of dry DMF was cooled to 0°C. BOP (143 mg, 0.31 mmol) and amine-allylsulfone **4c** (103 mg, 0.31 mmol) were then added to the solution. After stirring overnight at RT, the solvent was removed in vacuum. The residue was purified by column chromatography with 2% MeOH in $CHCl_3$ to afford 131 mg of the product **1d** as white solid in 77% yield. **^{1}H-NMR** (400 MHz, $CDCl_3$): δ = 1.34 - 1.36 (m, 2H), 1.54 - 1.76 (m, 4H), 2.20 (t, 2H, *J* = 7.58 Hz), 2.46 (s, 3H), 2.73 (d, 2H, *J* = 12.81 Hz), 2.91 (dd, 1H, *J* = 12.86 Hz, *J* = 4.88 Hz), 3.08 – 3.18 (m, 1H), 3.36 – 3.50 (m, 2H), 3.57 (t, 2H, *J* = 4.94 Hz), 3.69 (t, 2H, *J* = 4.44 Hz), 4.21 (s, 2H), 4.25 – 4.40 (m, 3H), 4.48 – 4.56 (m, 1H), 5.73 (s, 1H), 6.50 (s, 1H), 7.36 (d, 2H, *J* = 8.11 Hz), 7.78 (d, 2H, *J* = 8.27 Hz) ppm. **^{13}C-NMR** (100 MHz, $CDCl_3$): δ = 21.83, 25.83, 28.15, 28.27, 35.94, 39.25, 40.70, 55.65, 58.04, 60.26, 61.67, 64.40, 68.78, 70.08, 128.72, 128.98, 129.99, 133.78, 135.29, 145.31, 163.92, 165.09, 173.72 ppm. **LC-MS (ESI):** m/z = 554 $[M+H]^+$ (calcd. mass: 553.19, formula: $C_{25}H_{35}N_3O_7S_2$). **HR ESI-FTICR-MS:** m/z = 576.18066 $[M+Na]^+$, 592.15463 $[M+K]^+$ (calcd. exact mass: 576.18086 $[M+Na]^+$, 592.15480 $[M+K]^+$, formula: $C_{25}H_{35}N_3O_7S_2$).

Synthesis of 2-(6-(diethylamino)-3-(diethyliminio)-3H-xanthen-9-yl)-5-(N-(2-(2-((2-(tosylmethyl)acryloyl)oxy)ethoxy)ethyl)sulfamoyl)benzenesulfonate (1e)

Under argon atmosphere, amine-allyl sulfone **4c** (18.9 mg, 0.06 mmol) and lissamine rhodamine b sulfonyl chloride (50 mg, 0.09mmol, 1.5 equiv.) were dissolved in 1 mL of dry DMF and then DIEA (40 µL, 0.23 mmol, 4 equiv.) was added. After stirring for two days at RT, the solvent was evaporated in vacuum. The crude product was purified by column chromatography with 5% MeOH in DCM to afford 18.3 mg of the product **1e** as purple solid in

36% yield. **^{1}H-NMR** (400 MHz, $CDCl_3$): δ = 1.29 (t, 12H, *J* = 7.1 Hz), 2.42 (s, 3H), 3.27 (t, 2H, *J* = 10.8 Hz, *J* = 5.4 Hz), 3.48 - 3.68 (m, 12H), 4.14 – 4.22 (m, 4H), 5.82 (s, 1H), 6.53 (s, 1H), 6.65 (d, 2H, *J* = 2.4 Hz), 6.81 (dd, 2H, *J* = 9.5 Hz, *J* = 2.4 Hz), 7.21 (d, 1H, *J* = 7.9 Hz), 7.28 (d, 2H, *J* = 9.5 Hz), 7.33 (d, 2H, *J* = 8.0 Hz), 7.73 (d, 2H, *J* = 8.3 Hz), 7.98 (dd, 1H, *J* = 7.9 Hz, *J* = 1.9 Hz), 8.83 (d, 1H, *J* = 1.8 Hz) ppm. **^{13}C-NMR** (100 MHz, $CDCl_3$): δ = 12.74, 21.83, 43.33, 45.98, 57.90, 64.53, 68.98, 69.75, 95.74, 113.69, 114.54, 127.07, 127.58, 128.90, 129.02, 129.89, 129.93, 133.67, 133.83, 134.03, 135.38, 141.4, 145.16, 148.40, 155.68, 158.05, 159.22, 165.11 ppm. **LC-MS (ESI):** m/z = 868 $[M+H]^+$, 866 $[M-H]^-$ (calcd. mass: 867.25,formula:$C_{42}H_{49}N_3O_{11}S_3$). **HR MALDI-FTICR-MS:** m/z = 868.25948 $[M+H]^+$, 890.24132 $[M+Na]^+$, 906.21548 $[M+K]^+$, 1735.51034 $[2M+H]^+$, 1757.49435 $[2M+Na]^+$ (calcd. exact mass: 868.25544 $[M+H]^+$, 890.24214 $[M+Na]^+$, 906.21608 $[M+K]^+$, 1735.51312 $[2M+H]^+$, 1757.49507 $[2M+Na]^+$, formula: $C_{42}H_{49}N_3O_{11}S_3$), ε (572 nm, ACN/pB-Buffer pH = 8) = 87200 M^{-1} cm^{-1}.

3. Disulfide rebridging of somatostatin (SST).

(1) Synthesis and purification of ethinyl somatostatin 7a.

Somatostatin (1 mg, 0.6105 μmol) was dissolved in 500 μL of 50 mM PB (pH 7.8). TCEP (0.35 mg, 1.221 μmol) in 100 μL of 50 mM PB (pH 7.8) and allyl sulfone **1a** (0.50 mg, 1.221 μmol) in 900 μL of 50 mM PB pH 7.8 (sonication to dissolve) were added sequentially. The resulting mixture was incubated at RT for 24 h and purified by analytical HPLC using an MerckChroCART 125-4 Column with the mobile phase starting from 95 % solvent A (0.1 % TFA in H_2O) and 0% solvent B (0.1% TFA in ACN) (0-1min), raising to 20 % B at 5 min, further increasing to 45 % B at 17 min, reaching 70 % B at 19 min, finally returning 5 % B at 21 min and balanced the column for 2 min with a flow rate of 1 mL/min. The absorbance was monitored at 280 nm. The retention time for **7a** was 14.8 min. 0.6 mg of the product **7a** was obtained from lyophilisation in 49 % yield. **HR MALDI-FTICR-MS:** m/z = 2025.93150 $[M+H]^+$, 2047.91340 $[M+Na]^+$ (calcd. exact mass: 2025.93365 $[M+H]^+$, 2047.91559 $[M+Na]^+$, formula: $C_{95}H_{136}N_{18}O_{27}S_2$). For comparison, disulfide rebridging of somatostatin was also carried out in 50 mM PB (pH 7.8) or 50 mM PB containing 40 % ACN (pH 7.8) for 24 h. The reactions were analyzed by analytical HPLC.

Post-functionalization of ethynyl-somatostatin (7a) with a CuAAC

7a (1.2 mg, 0.59 μmol) was dissolved in 400 μL of demineralized water. Then, 40 uL azido coumarin **23** solution (0.24 mg, 1.18 μmol, in DMSO) was added. In another eppendorf tube, 20 uL $CuSO_4$ solution (0.19 mg, 1.18 μmol) and 20 uL sodium ascorbate solution (0.47 mg, 2.37 μmol) were mixed and intermediately added to **7a**. The resulting mixture was incubated at RT for 24 h and purified by analytical HPLC using an Agilent Eclipse XDB-C18 Column (9.4 x 250 mm, 5 μm) with the mobile phase starting from 95% solvent A (0.1% TFA in H_2O) and 0% solvent B (0.1% TFA in ACN) (0-1min), raising to 20 % B at 5 min, further increasing to 45% B at 17 min, reaching 95% B at 21 min, finally returning 5 % B at 24 min and balanced

the column for 2 min with a flow rate of 1 mL/min. The retention time for **coumarin-somatostatin** was 16.98 min. 1.02 mg of the product was obtained from lyophilisation in 77% yield. **HR MALDI-FTICR-MS:** m/z = 2228.96414 $[M+H]^+$ (calcd. exact mass: 2228.96674 $[M+H]^+$, formula: $C_{104}H_{141}N_{21}O_{30}S_2$). $\lambda_{Ex.}$ = 340 nm; $\lambda_{Em.}$ = 470 nm.

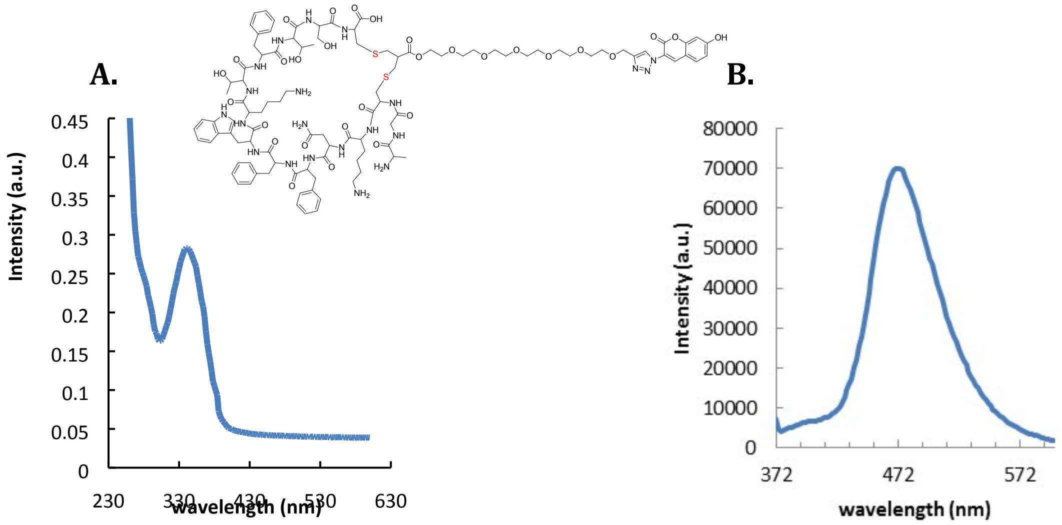

Fig. S1 Absorbance (A) and fluorescence spectra (B) of coumarin-somatostatin after HPLC purification. The spectra were recorded in water.

(2) Stability tests of rebridged somatostatin (7a) in Solutions with different pH

Ethinyl-SST (**7a**) was incubated in 50 mM pB-buffer solutions (c = 1 mg/mL) with physiological pH values (6, 7 and 8). Each solution was incubated for 2h, 8h, 25h, 49h and subsequently analyzed by analytical HPLC using an Agilent Eclipse XDB-C18 Column (9.4 x 250 mm, 5 μm) with the mobile phase starting from 95% solvent A (0.1% TFA in H_2O) and 0% solvent B (0.1% TFA in ACN) (0-1min), raising to 20 % B at 5 min, further increasing to 45% B at 17 min, reaching 95% B at 25 min, finally returning 5 % B at 27 min and balanced the column for 2 min with a flow rate of 1 mL/min. The injection volume was 50 uL for every single sample. The absorbance was monitored at 280 nm.

pH = 6

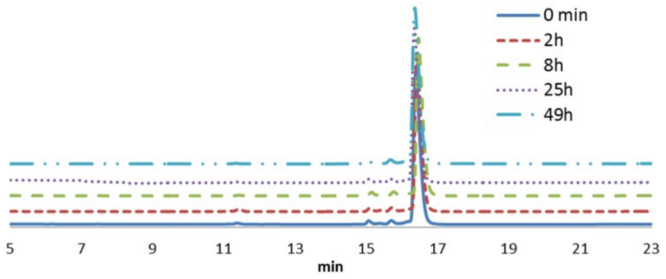

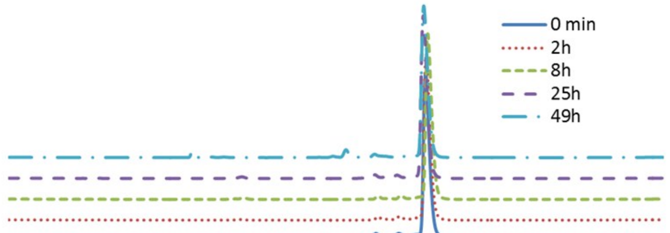

pH = 7

pH = 8

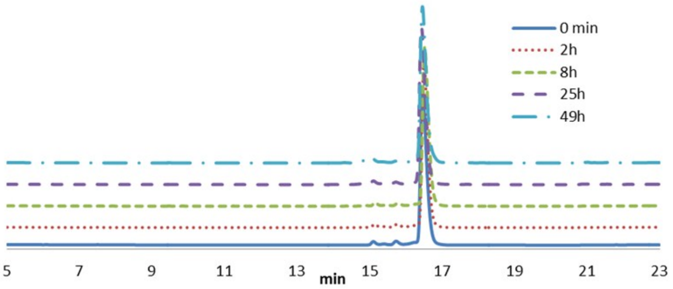

Fig. S2 HPLC profiles of 7a after incubation in 50 mM pB-buffer solutions with different physiologically relevant pH values. No difference was observed regarding peak position and peak height, indicating pH-stability of 7a for 49 h.

Fetal calf serum (FCS)

7a was dissolved in ultrapure water with 10% fetal calf serum (FCS) at a concentration of 1 mg/mL of peptide and incubated at 37 ^{o}C. 100 µL aliquots were taken at intervals of 0 h, 4 h, 8 h, 24 h and 48 h. Sample cleanup was then performed using silanized silica gel (1.5 cm in ultrapure water) packed in a 150 mm glass pipette to remove large proteins and the sample was eluted with 1 mL 60 % ACN with 0.1 % TFA, followed by 500 µL of 100 % ACN with 0.1% TFA. The solvents were then completely removed using speedvac and the samples reconstituted in 100 µL of 0.1 % formic acid in ultrapure water. Thereafter, the samples were diluted 20 times and 50 µg/mL of phenylalanine (Phe) was spiked as an internal standard.10% FCS solution was also subjected to the sample cleanup and preparation as a negative control. LC-MS analysis was performed on a Shimadzu LC-MS 2020 equipped with an electrospray ionsation source and a SPD-20A UV-Vis detector. (Shimadzu, Duisburg, Germany). Aliquots (40 µ L) were injected onto an Ascentis Express C18 column (150 x 4.6 mm, 2.7 µm). The column temperature was set at 40°C. The mobile phase consisted of 0.1% formic acid in water (B) and 0.1% formic acid in acetonitrile (A). The mobile phase was held for 8 mins at 95% B and changed to a linear gradient from 95% to 40% B from 8 to 18 mins, 40 to 5% B from 18 to 21 mins and hold at 5% B for 7 mins. The composition was changed back to 95% over 0.01 min and hold for 7 mins to for re-equilibration. The mobile phase flow rate was 0.4 mL/min. Identification of **7a** and hydrolysis products were performed simultaneously by UV-VIS detection at 254 nm and selective ion monitoring (SIM) of the [M + H] + at m/z =1013, 675 (**7a**);1724, 862, 575 (**7a** – ethynyl glycol), 321 (ethynyl glycol). The amount of **7a** in each sample was determined as a ratio of the integration of the chromatogram at 254 nm **7a** to the internal standard Phe. Background signals were

subtracted from the liquid chromatogram with a blank injection. Recovery of **7a** using the sample cleanup was determined to be 67%.

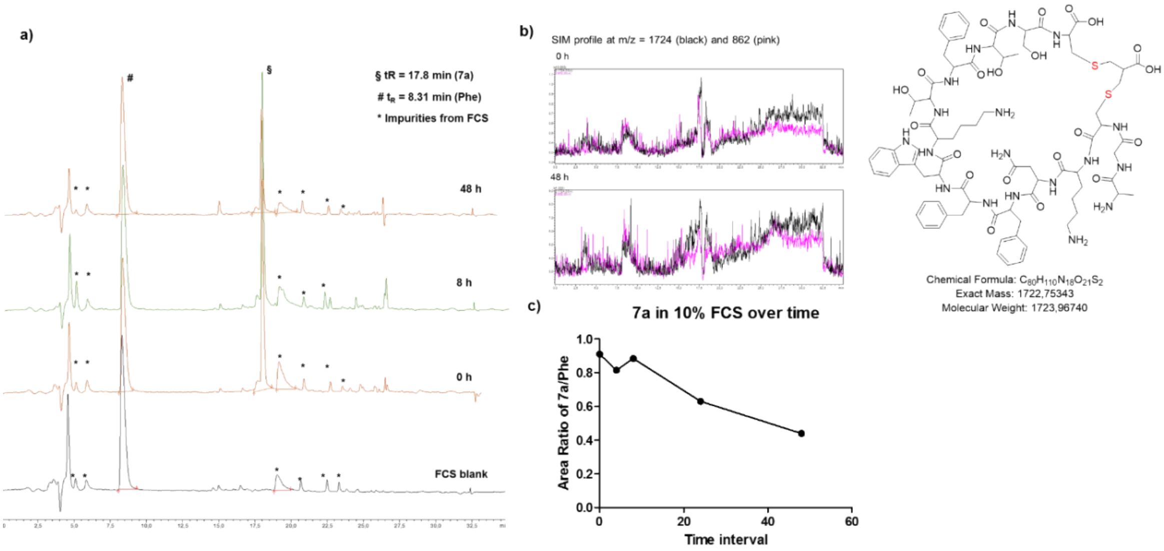

Fig. S3 (a) Liquid chromatogram of **7a** after incubation in 10% FCS at 37 °C at various time intervals. Minor peaks (*) in the chromatogram profiles are due to the background from 10 % FCS. 10% FCS solution was also analysed as a negative control. (b) No hydrolysis product was detected in the SIM profile at m/z = 1724 and 862 of **7a** even after incubation of 48 h in 10 % FCS indicating that **7a** is stable to hydrolysis under the test conditions. The decrease in **7a** is presumably due to adsorption by serum proteins present in FCS. (c) Plot of area ratio of **7a** against internal standard (Phe).

4. Disulfide rebridging of bovine insulin.

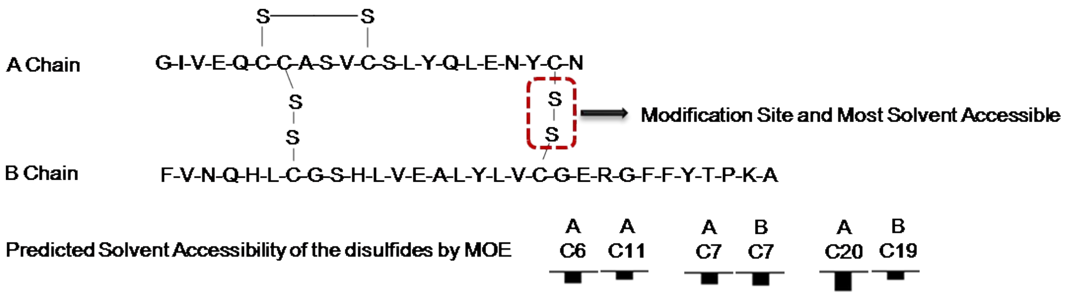

Fig. S4 The structure of bovine insulin and the solvent accessibility of the three disulfides predicted by MOE. The relative solvent accessibility of each cysteine residue is given as column length (“down” refers to accessible and “up” indicates buried inside).

Insulin (5 mg, 0.872 μmol) was dissolved in 500 μL of 0.01 N HCl. The pH was adjusted to around 7 using 1 N NaOH solution and 150 μL of 50 mM PB (pH 7.8) was added. Then, TCEP (0.3 mg, 1.05 μmol) in 50 μL of 50 mM PB (pH 7.8) and 50 μL of compound **1d** (1 mg, 1.8 μmol, 20 mg/ ml in DMSO) were added sequentially. The mixture was incubated at RT for

24 h and purified by Agilent 1260 HPLC using an Agilent Eclipse XDB-C18 Column (9.4 x 250 mm, 5 µm) with the mobile phase starting from 95% solvent A (0.1% TFA in water) and 5% solvent B (0.1% TFA in acetonitrile), raising to 30% B at 5 min, further increasing to 33% B at 10 min, keeping 33% B for 9 min, then reaching 95% B at 24 min, keeping 95% B for 1 min, finally returning 5% B at 30 min and balanced the column for 5 min with a flow rate of 4 mL/min. The absorbance was monitored at 280 nm and the retention time for BT-insu **8** was 12.3 min. 1.5 mg of BT-insu **8** was obtained from lyophilisation in 28% yield. In addition, 2 mg of native insulin was also recovered from HPLC purification. **HR ESI-MS**: m/z = 6131.96416 $[M+H]^+$ (calcd. mass: 6132.68).

Chymotrypsin digestion of BT-insu 8 for identification of the intercalation site

BT-insu (2 mg/mL) was digested with chymotrypsin (Sequencing Grade, Promega) at a ratio of 1:20 (chymotrypsin to BT-insu) in NH_4HCO_3 buffer (25 mM, pH 8). The mixture was shaken at 37℃ for 20 h on Eppendorf Thermomixer compact with a speed of 850 rpm. Following chymotryptic digest, resulting peptides from modified insulin were analyzed by LC-MS/MS. Peptide separation was performed on a Dionex U3000 uPLC with a Acclaim PepMap C18, 0.075x500 mm, 2 µm separation column, preceded with a Acclaim PepMap C18, 0.3*5 mm, 5 µm trap column (all ThermoScientific, Germany). A linear gradient from 15-70 % mobile phase B (50 % ACN, 30% Methanol, 5 % DMSO, 0.1 % formic acid) over 45 min. was applied. MS analysis was performed with an LTQ-OrbitrapVelos Pro (Thermo Scientific) and the $[^{13}C_1M+2H]^{2+}$ ion of the modified peptide (m/z = 830.867) was selected for fragmentation in the ion trap part of the instrument at a normalized collision energy of 35%.

Disulfide rebridging of bovine insulin using an excess of intercalator

Insulin (100 ug, 17.4 nmol) was dissolved in 10 µL of 0.01 N HCl. The pH was adjusted to around 7 using 1 N NaOH solution and 40 µL of 50 mM PB (pH 7.8) was added. Then, TCEP (47 ug, 0.16 umol) in 4,7 µL of 50 mM PB (pH 7.8) and 8,85 µL of compound **1a** (35 ug, 64 nmol, in AcN) were added sequentially. The mixture was incubated at RT for 24 h and characterized using MALDI-TOF analysis. **MALDI-TOF-MS**: m/z = 5731.7 $[M+H]^+$ (calcd. exact mass: 5730.6 $[M+H]^+$, formula: $C_{254}H_{377}N_{65}O_{75}S_6$); m/z = 6115.9 $[M+H]^+$ (calcd. exact mass: 6118.8 $[M+H]^+$, formula: $C_{273}H_{409}N_{65}O_{83}S_6$); m/z = 6505.1 $[M+H]^+$ (calcd. exact mass: 6507.0 $[M+H]^+$, formula: $C_{292}H_{441}N_{65}O_{91}S_6$), m/z = 6891.3 $[M+H]^+$ (calcd. exact mass: 6895.2 $[M+H]^+$, formula: $C_{311}H_{473}N_{65}O_{99}S_6$).

5. Disulfide rebridging of lysozyme (chicken egg white).

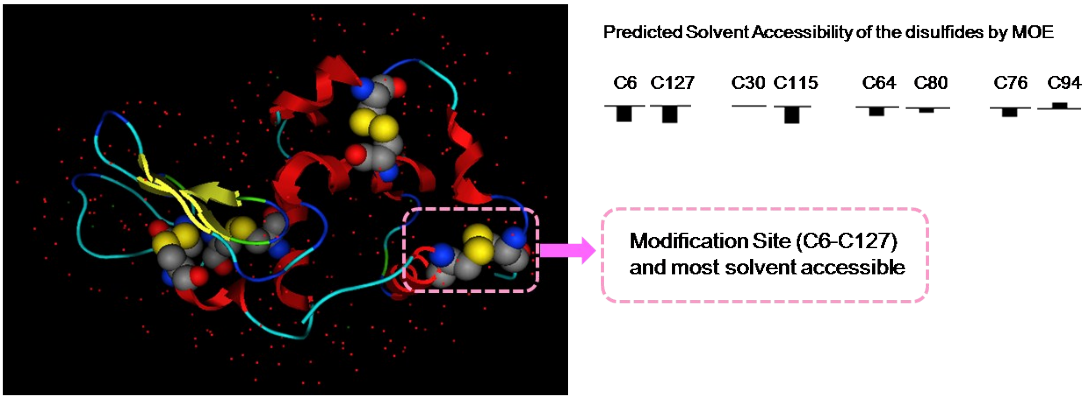

Fig. S5 The crystal structure of lysozyme from chicken egg and the solvent accessibility of the four disulfides predicted by MOE. The relative solvent accessibility of each cysteine residue is given as column length ("down" refers to accessible and "up" indicates buried inside).

Lysozyme (10 mg, 0.7 μmol) was dissolved in 4 mL of 50 mM PB (pH 7.8). TCEP (0.21 mg, 0.73 μmol) in 1 mL of 50 mM PB (pH 7.8) and compound **1b** (1 mg, 1.34 μmol) in 50 μL of DMSO were added sequentially. The resulting mixture was incubated at RT for 24 h and purified by FPLC (ÄKTA from GE) using a Hi Trap phenyl HP column (1 mL, from GE) with the mobile phase starting from 100% solvent A (0.1 M Na_2PO_4, 1.7M $(NH_4)_2SO_4$, pH 7.0) and 0% solvent B (0.1 M Na_2PO_4, pH 7.0) for 3 column volume (CV), raising to 40 % B in 1 CV, further increasing to 100% B in 18 CV, keeping 100 % for 5 CV, finally balanced the column 0 % B for 5 CV with a flow rate of 1 mL/min. The effluent peaks were monitored at 280 nm and 395 nm. 2 mg of C-Lyso **9** was obtained from lyophilisation in 19 % yield. In addition, 3 mg of native lysozyme was also recovered from FPLC purification. **HR ESI-MS**: m/z = 14897.33283 $[M+H]^+$ (calcd. mass: 14896.9).

Trypsin digestion of C-lyso 9 for identification of the intercalation site

Lysozyme or C-lyso **9** (2 mg/mL) was digested with trypsin (Sequencing Grade, Promega) at a ratio of 1:20 (trypsin to lysozyme) in 25 mM NH_4HCO_3 buffer. The mixture was shaken at 37℃ for 20 h on Eppendorf Thermomixer compact with a speed of 850 rpm. The peptide fragments were analyzed by HR MALDI-MS using α-Cyano-4-hydroxycinnamic acid (CHCA) as matrix (Fig. S6).

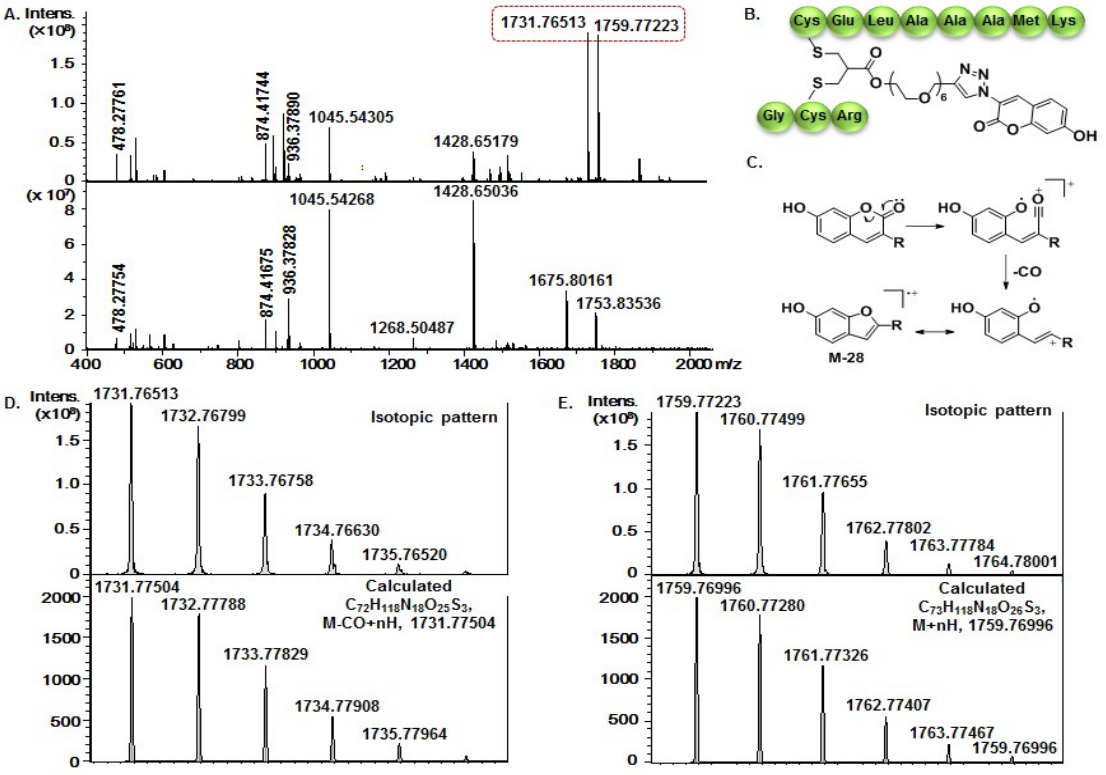

Fig. S6 A. The HR MALDI-MS for the tryptic digested C-Lyso **9** (up) and native lysozyme (bottom). The peptide fragment containing the rebridged disulfide is highlighted in red. B. The structure of the peptide fragment with the rebridged disulfide. C. The process of coumarin cleaved during mass measurement, resulting the reduced molecular mass (-CO, Δ m/z = 28). D. The isotopic pattern of the peptide fragment with cleaved coumarin (top) fits well with the calculated pattern (bottom). E. The isotopic pattern of the peptide fragment fits well with the calculated pattern (bottom).

The coumarin modified peptide fragment was sequenced by LC-MS/MS. It was separated using an Acclaim PepMap C18 0.075x20mm, 3μm trap column and Acclaim PepMap C18, 0.075x150mm, 2 μm column (both Thermo Fisher Scientific GmbH, Dreieich, Germany) with a linear gradient from 1-50% mobile phase B (76% ACN, 4% DMSO, 0.1% formic acid). MS analysis was performed with a Q Exactive (Thermo Scientific) and the $[M+3H]^{3+}$ ion (m/z = 587.2615) was used for fragmentation (CE 25, MS^2 resolution 70000).

6. **Circular dichroism (CD) of C-lyso 9 and lysozyme.**

Circular dichroism spectroscopy was conducted using an Applied Biophysics Chirascan spectropolarimeter and a JASCO J-810 spectropolarimeter using 0.2 cm cuvettes. A solution of lysozyme or C-Lyso **9** (19 μM) were placed in quartz cell, and the average of three scans from 175 to 300 nm was reported.

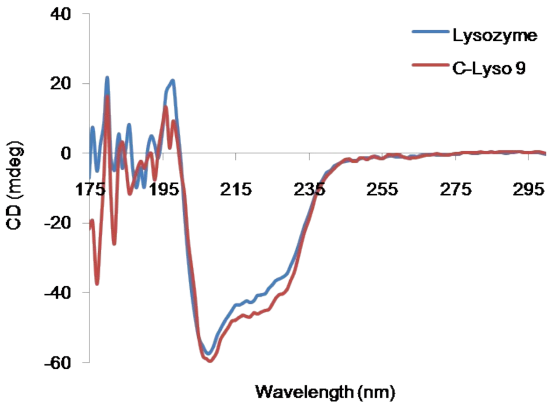

Fig. S7 The CD spectra of coumarin modified lysozyme C-Lyso **9** (red) and native lysozyme (blue).

7. Lysozyme activity assay.

10 mL of *Micrococcus lysodeikticus* cell suspension in potassium phosphate buffer (66 mM, pH 6.24) was prepare, so the A_{450} (absorbance at 450 nm) of the suspension is between 0.6-0.7 compared to the blank (potassium phosphate buffer). The A_{450} is monitored by Microplate Readers until the reading is constant at 25℃. 300 μL of the blank (potassium phosphate buffer), the control (0.42 μM of lysozyme) and the test (0.42 μM of C-lyso **9**) were added to a 96-well plate and their A_{450} were recorded for 6 min. The text was repeated for 3 times. The linear rate (ΔA_{450}/minute) was obtained for the blank, the control and the test (Fig. S6). The activity of lysozyme or C-Lyso **9** was calculated according to the equation.

units/mL = $\dfrac{\Delta A450/\min\ (Test) - \Delta A450/\min\ (Blank)}{0.001 * 0.3}$ units/nmol solid= $\dfrac{units/mL\ enzyme}{nmol\ solid/mL\ enzyme}$

Specification: 0.001 = ΔA_{450} as per the Unit Definition

0.3 = Volume (in milliliters) of enzyme solution

Unit Definition: One unit will produce a ΔA_{450} of 0.001 per minute at pH 6.24 at 25℃, using a suspension of Micrococcus lysodeikticus as substrate, in a 0.3 mL reaction mixture (96 well plate).

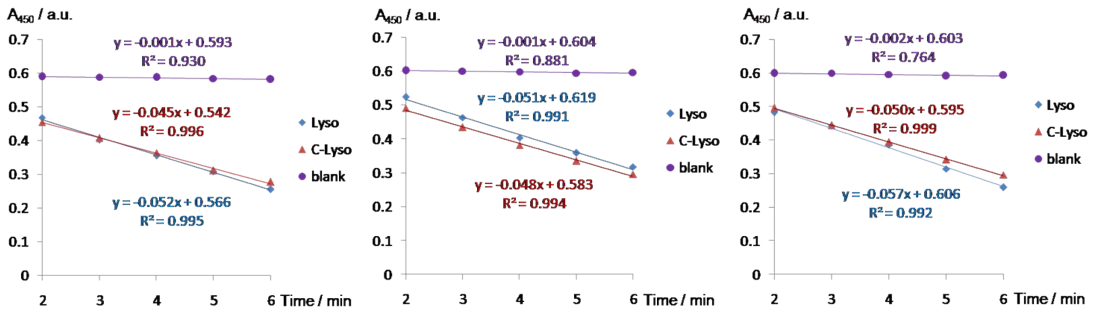

Fig. S8 A_{450} of 300 μl of C-Lyso, lysozyme and blank with Micrococcus lysodeikticus cell suspension in potassium phosphate buffer (66 mM, pH 6.24) were recorded for 6 min and their linear rates (ΔA_{450}/minute) were obtained. The measurement was repeated for 3 times.

After fitting the data ΔA_{450}/minute (from Fig. S8) into the equation, the catalytical efficiency of the C-Lyso **9** and lysozyme has been calculated in the table.

sample	units/nmol solid			average	STD	STD%
C-Lyso **9**	349.21	373.02	380.95	367.7	16.5	4.5
lysozyme	404.76	396.83	436.51	412.7	21.0	5.1

Table 1 The catalytical efficiency of the C-Lyso and lysozyme.

Therefore, the catalytical efficiencies of C-Lyso **9** and lysozyme were 367.7 ± 16.5 and 412.7 ± 21.0 units/nmol, respectively.

8. Allyl sulfones 4 as trifunctional building block for step-wise conjugation of thiol containing molecules by adjusting pH.

(1) Synthesis of the reagents.

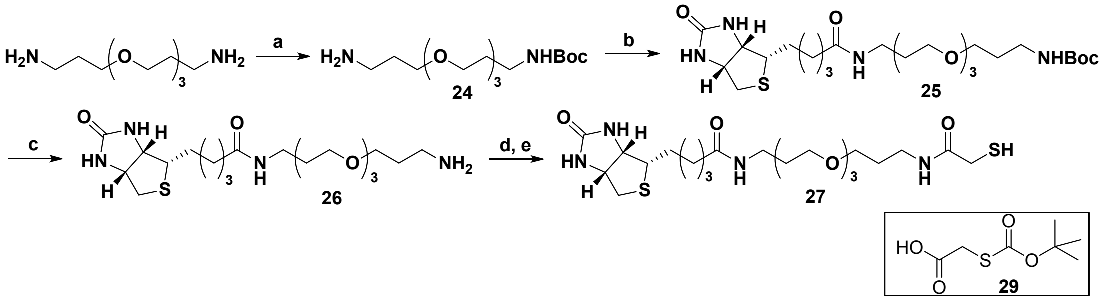

Scheme 2 The synthetic scheme of SH-biotin **27**. a. Boc_2O, DCM, 43%; b. Biotin, EDC, DMAP, anhydrous DMF, 72%; c.TFA, DCM, 95%; d. 27, HBTU, DIEA, DMF, 78%; e. TFA, DCM, 96%.

Synthesis of tert-butyl (3-(2-(2-(3-aminopropoxy)ethoxy)ethoxy)propyl) carbamate (24)[2]

A solution of 4,7,10-trioxoxa-1,13 -tridecanediamine (1 g, 1 ml) in DCM (15ml) was treated with di-*tert*-butyl dicarbonate (0.521ml) dropwise for 15 min. The mixture was stirred at RT for 12h. The solvent was removed, and the resulting yellow oil was purified by silica gel flash chromatography (10% MeOH in DCM containing 1% NH_4OH) to produce 313 mg of **24** as oil product in 43% yield. **^{1}H-NMR** (400MHz, $CDCl_3$): δ =1.39 (s, 9H), 1.72 (m, 4H), 2.79 (m, J = 6.6 Hz, 2H), 3.18 (m, 2H) 3.48-3.62 (m, 12H), 5.11 (s,2H) ppm; **^{13}C-NMR** (100MHz, $CDCl_3$): δ = 28.5, 29.6, 32.7, 38.4, 39.6, 69.6, 70.2, 70.6, 78.9, 156.1 ppm.

Synthesis of *tert*-butyl (15-oxo-19-((3a*S*,4*S*,6a*R*)-2-oxohexahydro-1*H*-thieno [3,4-*d*]imidazole-4-yl)-4,7,10-trioxa-14-azanonadecyl)carbamate (25)[5]

Biotin (254 mg, 1.04 mmol, 1 equiv.), 4-dimethylaminopyridine (DMAP, 14 mg, 124.8 μmol, 0.12 equiv.), 1-ethyl-3-(3-dimethylaminopropyl)carbodiimide (EDC, 400 mg, 1.24 mmol, 1.2 equiv.) and compound **24** (400 mg, 1.24 mmol, 1.2 equiv.) were dissolved in 6 mL of

anhydrous DMF under argon at 0℃. The reaction mixture was then warmed to RT and stirred overnight at RT. The mixture was purified by column chromatography with 8% methanol in chloroform to afford 492 mg of the product **25** in 72% yield. **^{1}H-NMR** (400 MHz, $CDCl_3$): δ = 1.34 (s, 11H), 1.57 – 1.68 (m, 8H), 2.09 (m, 2H), 2.65 (d, 1H, *J* = 12.8 Hz), 2.80 (d, 1H, *J* = 12.7 Hz), 3.04 – 3.12 (m, 3H), 3.22 (m, 2H), 3.44 – 3.54 (m, 12H), 4.21 (s, 1H), 4.41 (s, 1H), 5.10 (s, 1H), 6.13 (s, 1H), 6.71 (s, br, 1H), 6.77 (s, br, 1H) ppm. **^{13}C-NMR** (100 MHz, $CDCl_3$): δ = 25.70, 28.06, 28.30, 28.39, 28.95, 29.59, 35.99, 37.35, 38.35, 40.44, 55.73, 60.16, 61.72, 69.39, 69.60, 69.92, 70.07, 70.36, 70.40, 78.77, 156.00, 164.23, 173.23 ppm. **LC-MS**: m/z = 547 $[M+H]^+$, 569 $[M+Na]^+$, 447 $[M\text{-}Boc +H]^+$ (calcd. mass: 546.31, formula: $C_{25}H_{46}N_4O_7S$).

Synthesis of *N*-(3-(2-(2-(3-aminopropoxy)ethoxy)ethoxy)propyl)-5-((3a*S*,4*S*,6a*R*)- 2-oxohexahydro-1*H*-thieno[3,4-*d*]imidazol-4-yl)pentanamide (26)[5]

Compound **25** (400 mg, 0.73 mmol, 1 equiv.) was dissolved in 4 ml of DCM and trifluoroacetic acid (TFA, 1.67 g, 14.6 mmol, 20 equiv.) was added. The resulting mixture was stirred at RT overnight. The solvent and TFA were removed under vacuum to afford 310 mg of the product **26** in 95% yield. **^{1}H-NMR** (400 MHz, MeOH-d4): δ =1.44 (m, 2H), 1.55-1.79 (m, 6H), 1.93 (m, 2H), 2.21 (t, 2H, J = 7.4 Hz), 2.71 (d, 1H, J = 12.7 Hz), 2.93 (dd, 1H, J = 12.8 Hz, J = 5.0 Hz), 3.10 (t, 2H, J = 6.4 Hz), 3.19-3.27 (m, 3H), 3.51 (t, 2H, J = 6.1 Hz), 3.58-3.68 (m, 10H), 4.31 (m, 1H), 4.50 (m, 1H) ppm. **^{13}C-NMR** (100 MHz, MeOH-d4): δ = 26.88, 28.03, 29.50, 29.77, 30.48, 36.76, 37.65, 40.04, 41.04, 57.02, 61.61, 63.37, 69.68, 70.32, 70.99, 71.04, 71.07, 71.36, 166.07, 176.03 ppm. **LC-MS**: m/z = 447 $[M+H]^+$ (calcd. mass: 446.26, formula:$C_{20}H_{38}N_4O_5S$).

Synthesis of *ß*-boc-mercaptoacetic acid (29)[6]

In a 25 mL round bottom flask, *ß*-mercaptoacetic acid (150 µL, 2.17 mmol) was added to 3.2 mL of ^{t}BuOH/NaOH (1:1) at 0 °C. Then 550 µL of Boc_2O (2.38 mmol, 1.1 eq.) was added drop wise to the solution. The reaction mixture was stirred at RT overnight and the ^{t}BuOH was removed in vacuum. The pH of the solution was adjusted to three (citric acid) and the water phase was extracted with DCM (3x). The combined organic layers were dried over $MgSO_4$. The solvent was removed in vacuum to afford 300 mg of the product **29** as white solid in 72% yield. **^{1}H-NMR** (400 MHz, $CDCl_3$): δ = 1.50 (s, 9H), 3.64 (s, 2H) ppm. **^{13}C-NMR** (100 MHz, $CDCl_3$): δ = 28.27, 33.19, 86.42, 167.84, 174.74 ppm.

Synthesis of *N*-(1-mercapto-2-oxo-7,10,13-trioxa-3-azahexadecan-16-yl)-5-((3a*S*,4*S*,6a*R*) -2-oxohexahydro-1*H*-thieno[3,4-*d*]imidazol-4-yl)pentanamide (27)

Compound **29** (172 mg, 0.9 mmol, 2 equiv.) was dissolved in 2 mL of anhydrous DMF under argon. Then *N,N,N',N'*-tetramethyl-O-(1H-benzotriazol-1-yl)uronium hexafluorophosphate (HBTU, 545 mg, 1.43 mmol, 3.2 equiv.) and *N,N*-diisopropylethylamine (DIEA, 296 µl, 1.79 mmol, 4 equiv.) were added to this solution at 0℃. The reaction mixture was stirred for 10 min

before compound **26** (200 mg, 0.45 mmol, 1 equiv.) in 1 mL of anhydrous DMF was added. The resulting mixture was stirred overnight at RT and the solvent was removed under high vacuum. The product was dissolved in $CHCl_3$ and washed with $NaHCO_3$ (2x), brine and dried over anhydrous Na_2SO_4. The solvent was evaporated under vacuum and the crude product was purified by column chromatography with 5% MeOH in $CHCl_3$ to afford 217 mg of the product **27** in 78% yield. **^{1}H-NMR** (400 MHz, $CDCl_3$): δ = 1.39 - 1.47 (m, 11H), 1.62 – 1.78 (m, 8H), 2.17 (t, 2H, *J* = 7.5 Hz), 2.72 (d, 1H, *J* = 12.8 Hz), 2.87 (dd, 1H, *J* = 4.8 Hz, *J* = 12.8 Hz), 3.06 – 3.141 (m, 1H), 3.27 – 3.35 (m, 4H), 3.44 (m, 2H), 3.50 – 3.63 (m, 12H), 4.29 (m, 1H), 4.49 (m, 1H), 5.79 (s, 1H), 6.49 (s, 1H), 6.75 (s, br, 1H), 6.97 (s, br, 1H) ppm. **^{13}C-NMR** (100 MHz, $CDCl_3$): δ = 25.79, 28.19, 28.24, 28.35, 29.07, 29.11, 34.81, 36.10, 37.70, 27.97, 40.62, 55.77, 60.31, 61.92, 69.59, 69.92, 70.09, 70.26, 70.49, 70.54, 86.07, 164.11, 168.29, 168.62, 173.38 ppm. **LC-MS**: m/z = 621 $[M+H]^+$, 521 $[M\text{-}Boc+H]^+$ (calcd. mass: 620.29, formula: $C_{27}H_{48}N_4O_8S_2$) This compound was used without further characterization. Compound **27** (210 mg, 0.34 mmol, 1 equiv.) was dissolved in 3 ml of DCM and trifluoroacetic acid (TFA, 771 mg, 6.76 mmol, 20 equiv.) was added. The resulting mixture was stirred at RT overnight. The solvent and TFA were removed under vacuum to afford 170 mg of the product **28** in 96% yield. **^{1}H-NMR** (400 MHz, $CDCl_3$): δ = 1.40 (m, 2H), 1.58 – 1.81 (m, 8H), 2.03 (t, 1H, *J* = 8.8 Hz), 2.16 (t, 2H, J = 7.6 Hz), 2.71 (m, 1H), 2.87 (dd, 1H, *J* = 4.9 Hz, *J* = 12.8 Hz), 3.10 (m, 1H), 3.18 (d, 2H, *J* = 8.8 Hz), 3.26 – 3.37 (m, 4H), 3.50 – 3.63 (m, 12H), 4.28 (m, 1H), 4.48 (m, 1H), 5.91 (s, 1H), 6.64 (s, 1H), 6.76 (t, 1H, *J* = 5.5 Hz), 7.39 (s, br, 1H) ppm. **^{13}C-NMR** (100 MHz, $CDCl_3$): δ = 25.83, 28.18, 28.35, 28.38, 28.88, 29.11, 36.13, 37.61, 38.4, 40.63, 55.84, 60.28, 61.88, 69.83, 70.05, 70.07, 70.21, 70.49, 70.51, 164.21, 169.80, 173.40 ppm. **HR MALDI-FTICR-MS:** m/z = 521.24593 $[M+H]^+$, 1039.46873 $[M_2+H]^+$ (calcd. exact mass: 521.24620 $[M+H]^+$, 1039.46948 $[M_2+H]^+$, formula: $C_{22}H_{40}N_4O_6S_2$).

(2) LC-MS study for the model reaction.

Step 1: Conjugation of L-cysteine at pH 6 and 8.

To a solution of 233.3 µL of PB (50 mM PB, 10 mM EDTA, pH 6 or 8), 60µL of compound **4a** (5 mg/mL in ACN, 1 equiv) and 6.7 µL of L-cysteine (20 mg/ml in MiliQ H_2O, 2 equiv) were added sequentially. These mixtures were shaken at RT on Eppendorf Thermomixer compact with a speed of 850 rpm. At 0 min, 5 min, 10 min, 15 min, 20 min, 40 min, 1 h, 3 h, 6 h, and 24 h, 10 µL was taken and quenched with 990 µL of 0.1% TFA in MiliQ H_2O. The solutions were stored at -20℃ before LC-MS study. LC-MS analysis was performed on a Shimadzu LC-MS 2020 equipped with an electrospray ionisation source and a SPD-20A UV-Vis detector (Shimadzu, Duisburg, Germany). Aliquots (10 µL) were injected into Ascentis Express C18 (150 x 4.6 mm, 2.7 µm) (Supelco). The column temperature was set at 40°C. The mobile phase consisted of 0.1% formic acid in water (A) and 0.1% formic acid in acetonitrile (B). The gradient was started from 5% B, balanced at 5% B for 8 min, increased to 60% B in 10 min,

further increased to 95% B in 3 min, held at 95% B for 7 min, then immediately stepped back down to 5% and re-equilibrated for 7 min. The mobile phase flow rate was 0.4 mL/min.

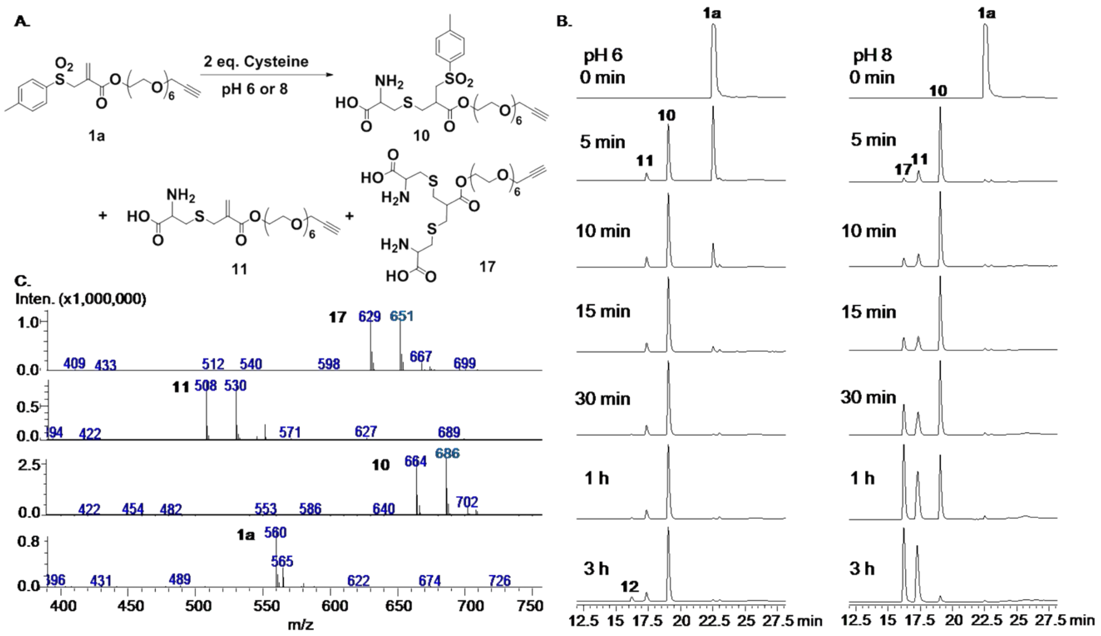

Fig. S9 A. The model of allyl sulfone **1a** reacted with 2 eq. cysteine at pH 6 or 8. B. The MS chromatogram in the selective ion mode (SIM) for this model reaction at pH 6 (left) or pH 8 (right). C. The MS spectra corresponding to the peaks in the MS chromatogram. **17**: m/z = 629 $[M+H]^+$, 651 $[M+Na]^+$ (chemical formula: $C_{25}H_{44}N_2O_{12}S_2$, calcd.mass: 628.23); **11**: m/z = 508 $[M+H]^+$, 530 $[M+Na]^+$ (chemical formula: $C_{22}H_{37}NO_{10}S$, calcd.mass: 507.21); **10**: m/z =664 $[M+H]^+$, 686 $[M+Na]^+$ (chemical formula: $C_{29}H_{45}NO_{12}S_2$, calcd. mass: 663.24); **1a**: m/z = 560 $[M+H_2O]^+$, 565 $[M+Na]^+$ (chemical formula: $C_{26}H_{38}O_{10}S$,calcd.mass: 542.22).

Step 2: Conjugation of GSH at pH 6 and 8.

Compound **4a** (14.5 mg, 26.7 μmol, 1 equiv) were dissolved in 150 μL of ACN and 440 μL of PB (50 mM, pH 6) was added. Thereafter, 30 μL of L-Cys (200 mg/ml in H_2O, adjust pH around 6, 2 equiv) was added and the resulting mixture was shaken at RT for 1h on Eppendorf Thermomixer compact with a speed of 850 rpm. The mixture was purified by Prep HPLC using an Atlantis Prep OBD T3 Column (19x100 mm, 5 μm). 13 mg of the product **10** was isolated in 75% yield. Compound **10** (0.06 mg, 90.5 nmol, 1 equiv) and GSH (0.278 mg, 905 nmol, 10 equiv) were dissolved in 60 μL of PB (50 mM, pH 6 or 8). The reaction mixture were shaken at RT and 10 μL of the mixture was taken to be quenched with 540 μL MeOH containing 0.18% formic acid after 1 h, 6 h, 9 h and 24 h respectively. These solutions were stored at -20℃ before LC-MS study. LC-MS analysis was performed on a Shimadzu LC-MS 2020 equipped with an electrospray ionisation source and a SPD-20A UV-Vis detector (Shimadzu, Duisburg, Germany). Aliquots (10 μL) were injected onto Ascentis Express C18 (150 x 4.6 mm, 2.7 μm) (Supelco). The column temperature was set at 40°C. The mobile phase consisted of 0.1% formic acid in water (A) and 0.1% formic acid in acetonitrile (B). The gradient was started from 0% B, balanced at 0% B for 5 min, increased to 25% B in 3 min,

held at 25% for 7 min, then increased to 50% B in 1 min, further increased to 70% B in 5 min, finally increased to 100% B in 1 min, held at 100% B for 5 min, then immediately stepped back down to 0% in 1 min and re-equilibrated for 4 min. The mobile phase flow rate was 0.4 mL/min.

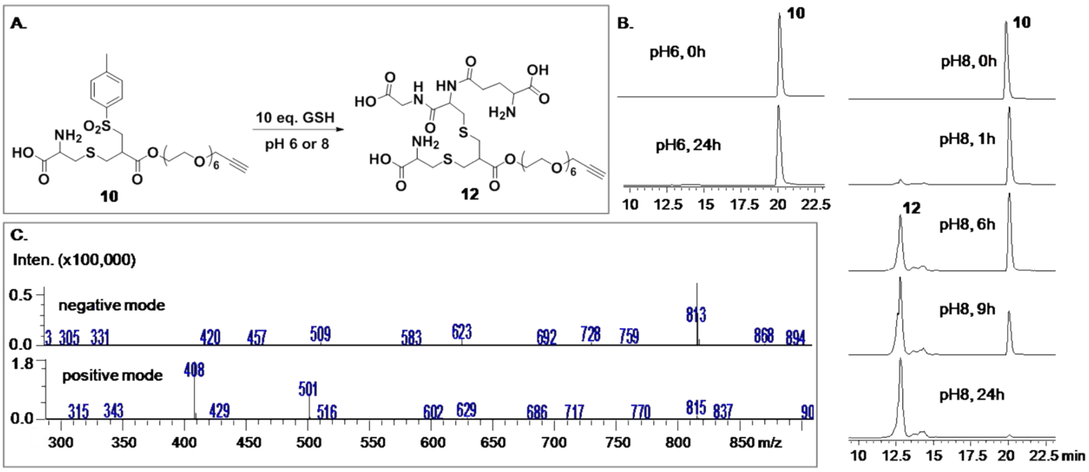

Fig. S10 A. The second step of the model reaction. B. The MS chromatogram in the selective ion mode (SIM) for this model reaction. C. The MS spectra of **12** in LC-MS. m/z = 813 $[M-H]^-$, 408 $[M+2H]^{2+}$ (chemical formula: $C_{32}H_{54}N_4O_{16}S_2$, calcd. mass: 814.30).

(3) Expression of GFP containing a single cysteine mutation.

Native GFP sequence was inserted to pET-25b(+) vector as describe before.[3] Gly51 was mutated to Cysteine using Q5 site-directed mutagenesis kit (New England Biolabs). Mutated protein was expressed in BL21(DE3) E. Coli cells under 25 °C for 4 hrs. mGFP (G51C) was purified by Ni-NTA affinity column, desalted and lyophilized. The sequence of mGFP (G51C) was shown below (single accessible cysteine is highlighted):

MSKGEELFTG VVPILVELDG DVNGHKFSVS GEGEGDATYG KLTLKFICTT CKLPVPWPTLVTTFSYGVQC FSRYPDHMKQ HDFFKSAMPE GYVQERTIFF KDDGNYKTRA EVKFEGDTLVNRIELKGIDF KEDGNILGHK LEYNYNSHNV YIMADKQKNG IKVNFKIRHN IEDGSVQLADHYQQNTPIGD GPVLLPDNHY LSTQSALSKD PNEKRDHMVL LEFVTAAGIT HGMDELGVVGLVPRGSHMGA GPGWPHHHHH H

(4) Allyl sulfone 1e for step-wise conjugation of SH-biotin and SH-GFP by increasing the pH.

Step 1: Conjugation of SH-biotin at pH 6.

A solution of rhodamine-allyl sulfone **1e** (10 mg, 11.52 μmol, 1 equiv.) and SH-biotin **28** (7 mg, 13.82 μmol, 1.2 equiv.) in 100 μl of DMF was dissolved in 1 ml of PB (50 mM, pH 6). The reaction mixture was stirred at RT for 1h and the solvent was removed by lyophilization. The mixture was purified by column chromatography using 10% MeOH in $CHCl_3$ with 1% CH_3COOH to afford 14 mg of the products **13** and **14** in 98% yield. The products were characterized by LC-MS. LC-MS: m/z = 617 $[M+2H]^{2+}$ (**13**, calcd. mass: 1231.47), m/z = 695 $[M+2H]^{2+}$ (**14**, calcd. mass: 1387.49).

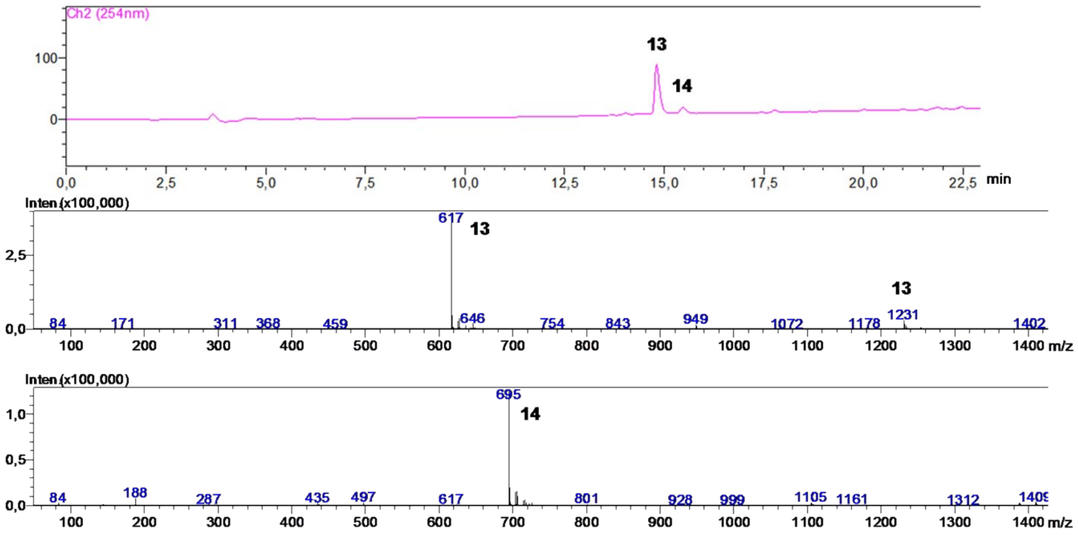

Fig. S11 The LC-MS spectra of the product synthesized by coupling SH-biotin on allyl sulfone **1e** at pH 6.

Step 2: Conjugation of SH-GFP at pH 8.

SH-GFP (0.5 mg, 17.08 nmol, 1 eq.) was dissolved in 450 μl of PB (25 mM, pH 8). Then the products **13** and **14** from the first step (0.4 mg, 341.5 nmol, 20 eq.) in 50 μl of DMF were added. The resulting mixture was shaken overnight on Eppendorf Thermomixer compact with a speed of 850 rpm. The mixture was purified by size exclusion chromatography using Sepharose G-25 matrix and 0.47 mg of the product **15** was obtain after lyophilisation in 90% yield. As control experiment, SH-GFP reacted with the products 13 and 14 from the first step in 25 mM PB (pH 6) and 0.48 mg of the product **16** was obtain after lyophilisation in 96 % yield. The product **15** and **16** were characterized by absorbance and emission spectra, SDS gel electrophoresis and biotin detection *via* western blotting. The absorbance of the conjugate **15** at 492 nm and 572 nm were 0.5326 and 0.4632, and the extinction coefficient of GFP and lissamine rhodamine b were 55,000 $M^{-1}cm^{-1}$ (in water, Ref. 4) and 87,200 $M^{-1}cm^{-1}$ (Fig. S13) respectively. Therefore, the labeling efficiency of SH-GFP was 55%.

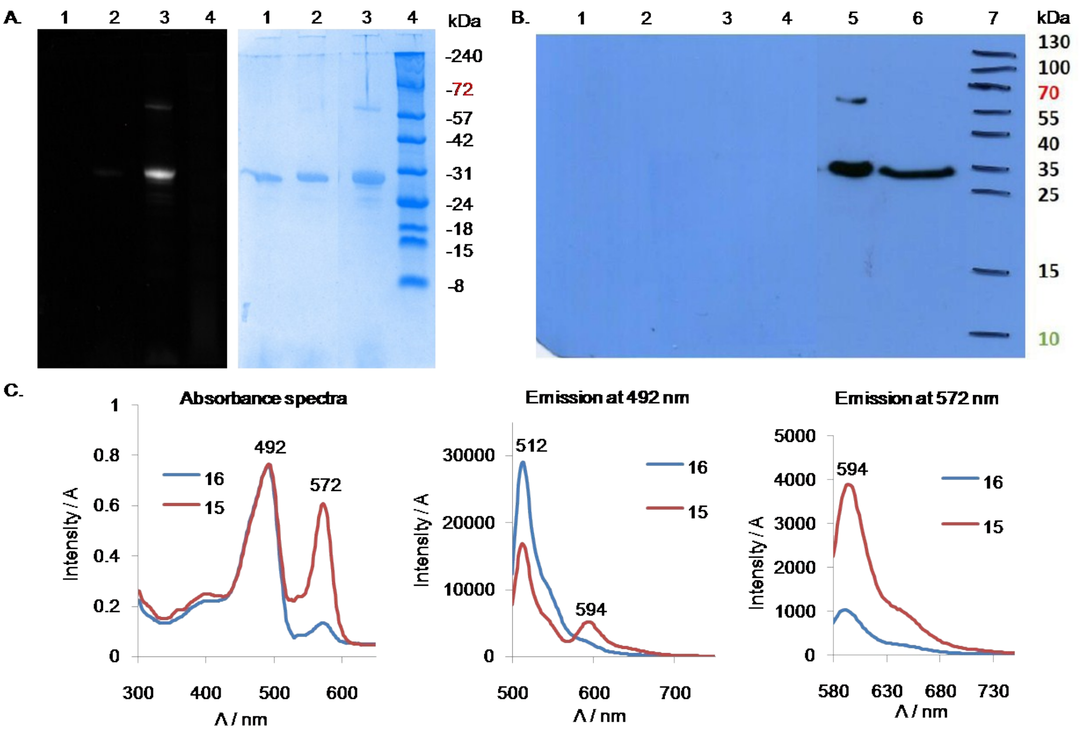

Fig. S12 A. SDS-PAGE analysis using fluorescence visualization (left) and Coomassie staining (right), lane 1: SH-GFP (1.5 µg), lane 2: conjugate **16** (1.5 µg), lane 3: conjugate **15** (1.5 µg), lane 4: protein marker. B. Detection of biotin group *via* western blotting, lanes 1: SH-GFP (600 ng), lane 2: SH-GFP (300 ng), lane 3: conjugate **16** (600 ng), lane 4: conjugate **16** (300 ng), lane 5: conjugate **15** (600 ng), lane 6: conjugate **15** (300 ng), lane 7: protein marker, C. The absorbance and emission spectra of the conjugates **15** and **16**.

Determination of biotin group on the GFP.

(1). Western blot

Two different amounts of SH-GFP, conjugate **16** and **15** (600 ng and 300 ng respectively) were separated by SDS-PAGE and then transferred to a nitrocellulose membrane *via* Western blotting. After blocking the membrane for 1 h with 5 % dry milk in PBS containing 0.1 % Tween-20, the successful biotin labeling was detected with streptavidin-peroxidase by using a chemiluminescence (ECL) system according to the manufacturer's instructions.

(2). Immobilization of the conjugate 15 on streptavidin agarose.

200 µl of streptavidin agarose (invitrogen) was put in the eppendorf tube and the solvent was removed by pipette. Thereafter, 60 µl of the conjugate **15** (0.67 mg/ml in MiliQ H_2O) were added and incubated for 30 min. The streptavidin agarose was centrifuged down in 2 min (1000 x g). The absorbance of the supernatant (40 µl, λ = 574 nm) was measured in Greiner 384 flat bottom transparent plate and the reading was 0.2683 ± 0.0020. As control, the absorbance of 40 µl of the conjugate **15** (0.67 mg/ml in MiliQ H_2O) and 40 µl of MiliQ H_2O were also recorded as 0.0564 ± 0.0002 and 0.0350 ± 0.0002 respectively. Therefore, there is

100%-(0.0564-0.0350)/(0.2683-0.035)x100% = 91% of the conjugate **15** were immobilized on the streptavidin agarose.

Determination of the extinction coefficient of lissamine rhodamine b intercalator (1e) in water.

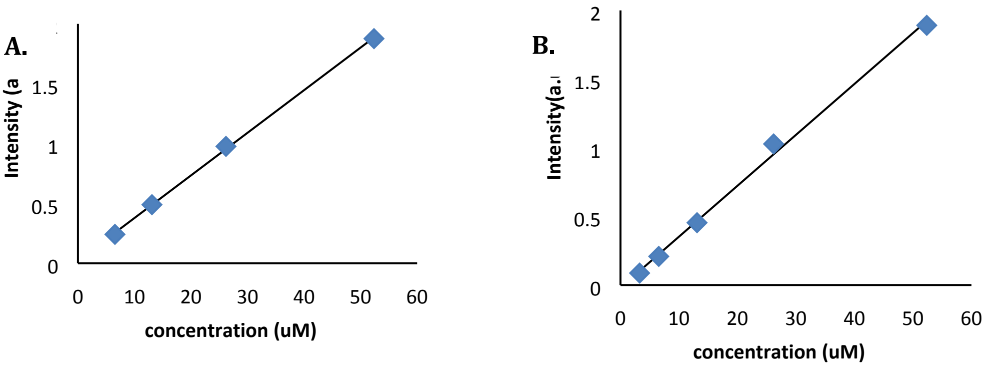

Fig. S13 Determination of the extinction coefficient ε. The absorbance at 572 nm was monitored by Microplate Readers at 25℃. Using lambert beers law, extinction coefficients were determined as 87200 $M^{-1}\cdot cm^{-1}$ for 1e **(A)** and 90800 $M^{-1}\cdot cm^{-1}$ for lissamine rhodamine sulfonýl chloride **(B)** in ACN, pB-Buffer (pH = 8, 50 mM) mixture (50:50).

9. (HR)-MS spectra

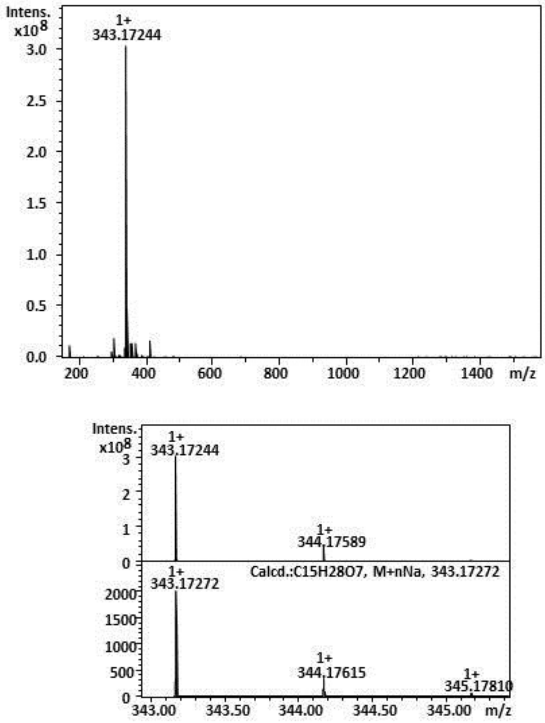

Fig. S14 HR ESI-FTICR-MS spectrum of propargyl-hexaethylene glycol (**18**): m/z = 343.17244 $[M+Na]^+$ (calcd. exact mass: 343.17272 $[M+Na]^+$, formula: $C_{15}H_{28}O_7$).

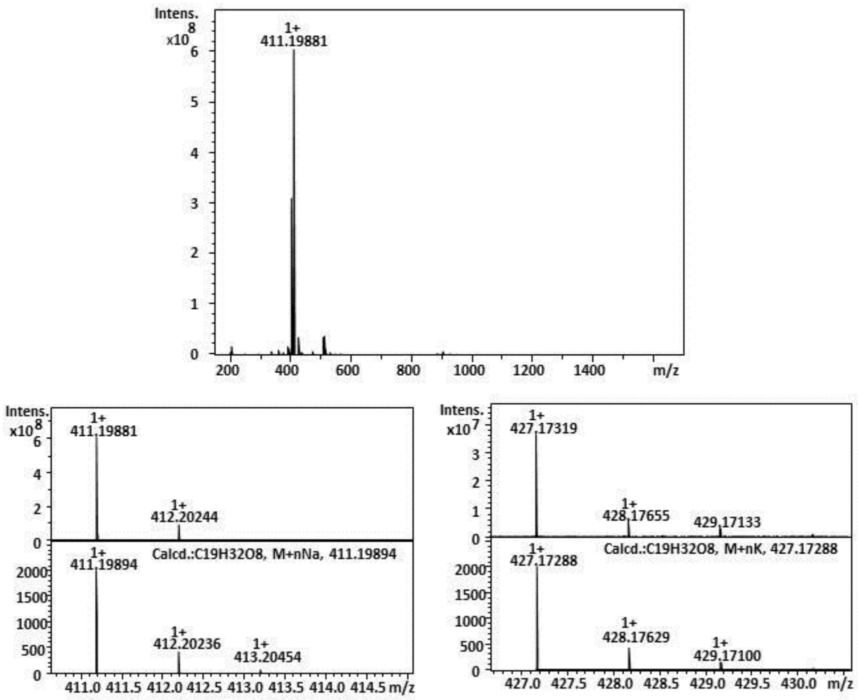

Fig. S15 HR ESI-FTICR-MS spectrum of methacryl-propargyl-hexaethylene glycol (**19**): m/z = 411.19881 [M+Na]$^+$, 427.17319 [M+K]$^+$ (calcd. exact mass: 411.19894 [M+Na]$^+$, 427.17288 [M+K]$^+$, formula: $C_{19}H_{32}O_8$).

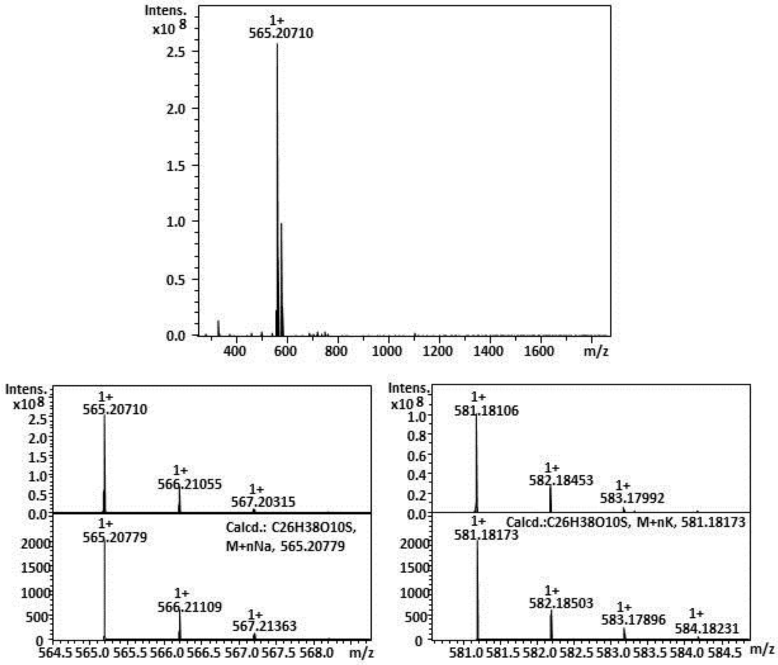

Fig. S16 HR MALDI-FTICR-MS spectrum of 3,6,9,12,15,18-hexaoxahenicos-20-yn-1-yl 2-(tosylmethyl)acrylate (**1a**): m/z = 565.20710 [M+Na]$^+$, 581.18106 [M+K]$^+$ (calcd. exact mass: 565.20779 [M+Na]$^+$, 581.18173 [M+K]$^+$, formula: $C_{26}H_{38}O_{10}S$).

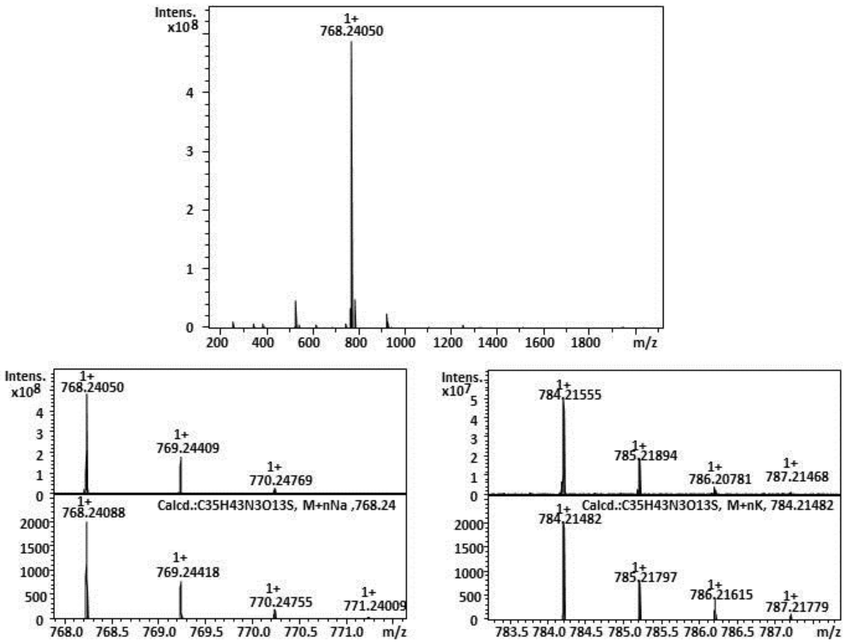

Fig. S17 HR ESI-FTICR-MS spectrum of 1-(1-(7-hydroxy-2-oxo-2H-chromen-3-yl)-1H-1,2,3-triazol-4-yl)-2,5,8,11,14,17-hexaoxanonadecan-19-yl 2-(tosylmethyl)acrylate (**1b**)**:** m/z = 768.24050 $[M+Na]^+$, 784.21555 $[M+K]^+$ (calcd. exact mass: 768.24088 $[M+Na]^+$, 784.21482 $[M+K]^+$, formula: $C_{35}H_{43}N_3O_{13}S$).

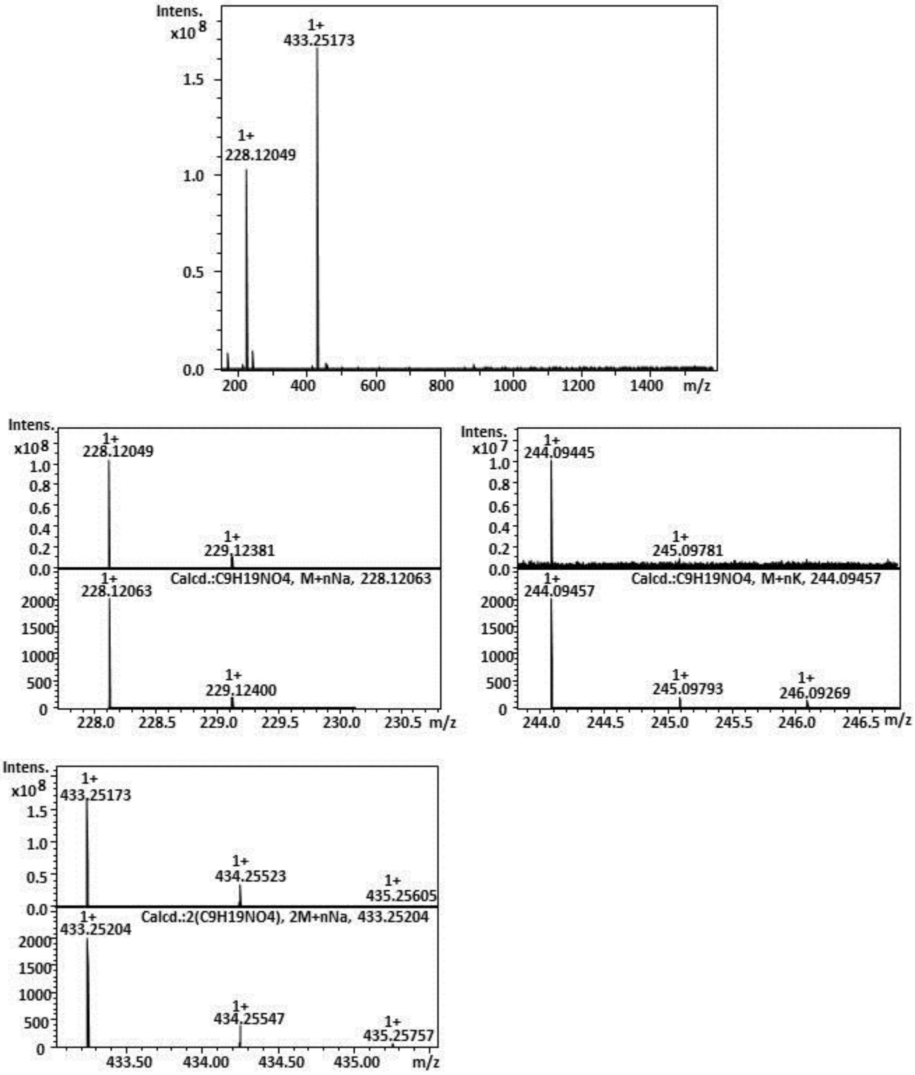

Fig. S18 HR ESI-FTICR-MS spectrum of *tert*-butyl (2- (2-hydroxyethoxy) ethyl)carbamate (**20**)**:** m/z = 228.12049 $[M+Na]^+$, 244.09445 $[M+K]^+$, 433.25173 $[2M+Na]^+$ (calcd. exact mass: 228.12063 $[M+Na]^+$, 244.09457 $[M+K]^+$, 433.25204 $[2M+Na]^+$, formula: $C_9H_{19}NO_4$).

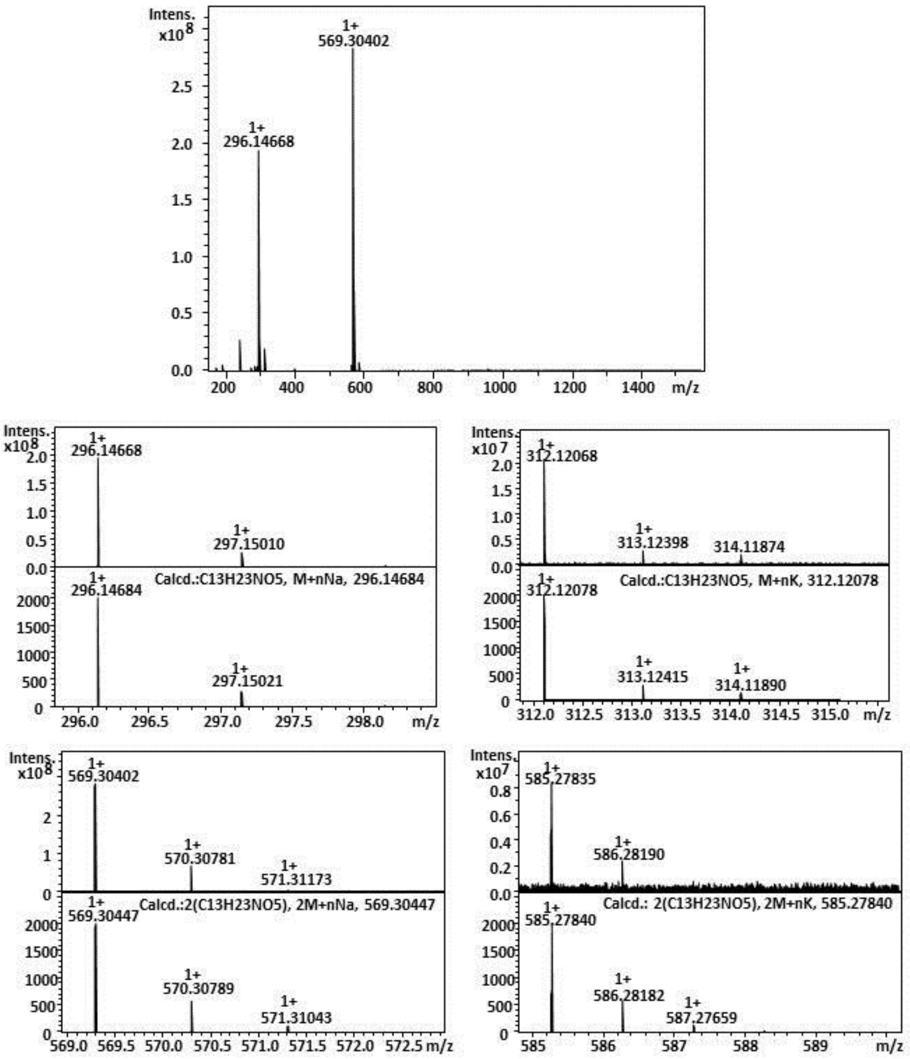

Fig. S19 HR ESI-FTICR-MS spectrum of 2-(2-((tert-butoxycarbonyl) amino) ethoxy)ethyl methacrylate (**21**)**:** m/z = 296.14668 $[M+Na]^+$, 312.12068 $[M+K]^+$, 569.30402 $[2M+Na]^+$, 585.27835 $[2M+K]^+$ (calcd. exact mass: 296.14684 $[M+Na]^+$, 312.12078 $[M+K]^+$, 569.30447 $[2M+Na]^+$, 585.27840 $[2M+K]^+$, formula: $C_{13}H_{23}NO_5$).

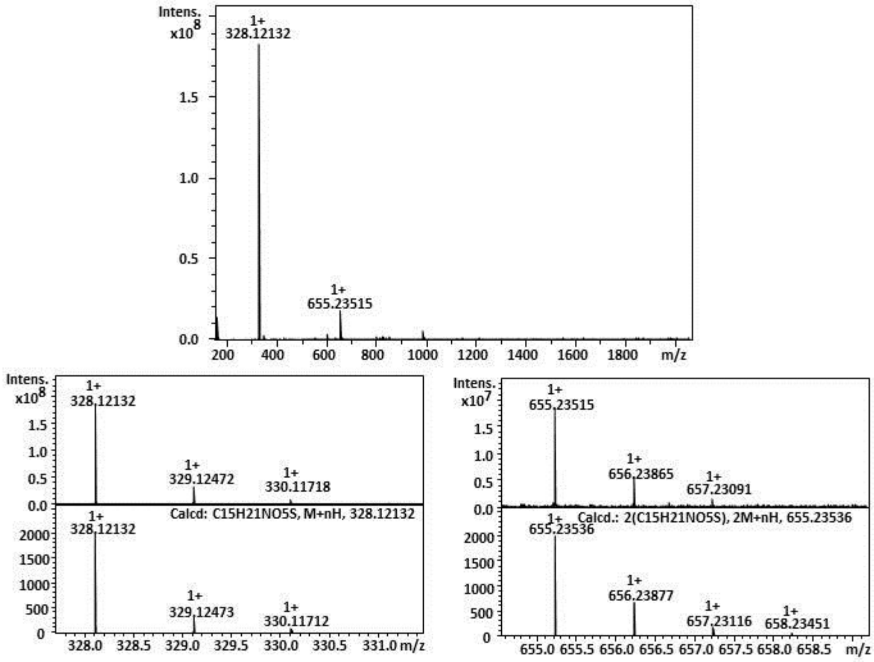

Fig. S20 HR ESI-FTICR-MS spectrum of 2-(2-aminoethoxy)ethyl 2-(tosylmethyl)acrylate (**1c**)**:** m/z = 328.12132 $[M+H]^+$, 655.23515 $[2M+H]^+$ (calcd. exact mass: 328.12132 $[M+H]^+$, 655.23536 $[2M+H]^+$, formula: $C_{15}H_{21}NO_5S$).

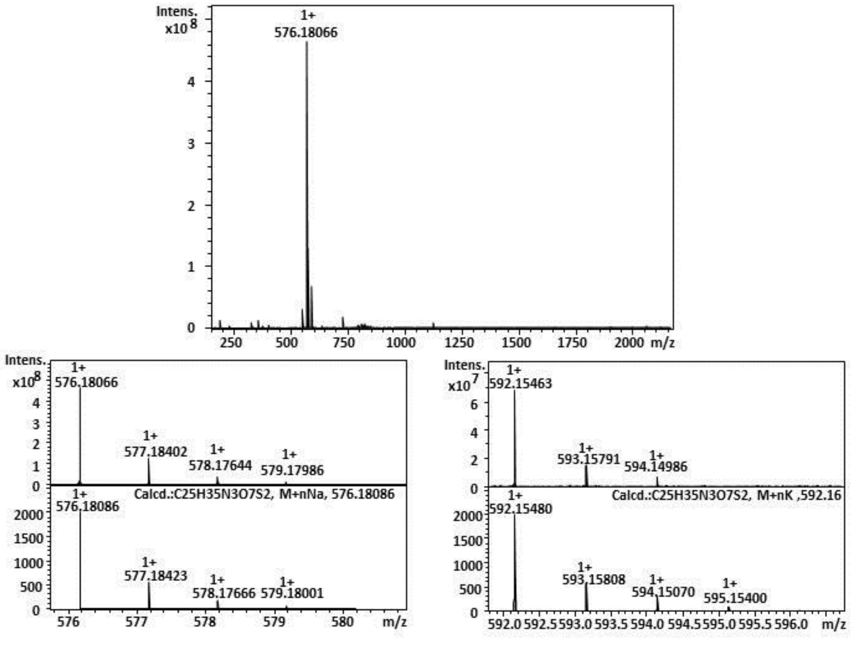

Fig. S21 HR ESI-FTICR-MS spectrum of 2-(2-(5-((3aS,4S,6aR)-2-oxohexahydro-1H-thieno [3,4-d]imidazol-4-yl) pentanamido)ethoxy)ethyl 2-(tosylmethyl)acrylate (**1d**)**:** m/z = 576.18066 $[M+Na]^+$, 592.15463 $[M+K]^+$ (calcd. exact mass: 576.18086 $[M+Na]^+$, 592.15480 $[M+K]^+$, formula: $C_{25}H_{35}N_3O_7S_2$).

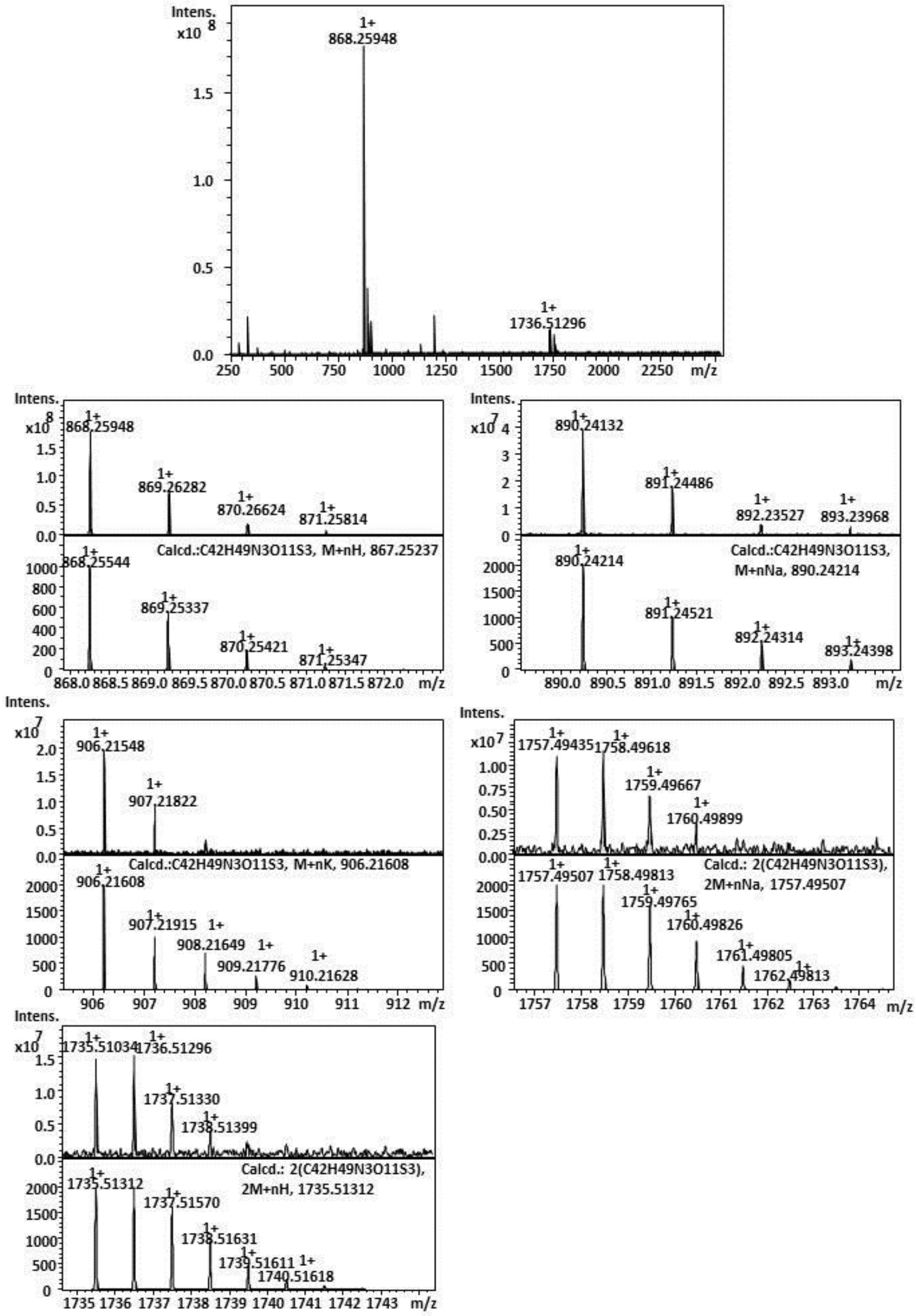

Fig. S22 HR MALDI-FTICR-MS spectrum of 2-(6-(diethylamino)-3-(diethyliminio)-3H-xanthen-9-yl)-5-(N-(2- (2-((2-(tosylmethyl)acryloyl)oxy)ethoxy)ethyl)sulfamoyl)benzenesulfonate (**1e**)**:** m/z = 868.25948 $[M+H]^+$, 890.24132 $[M+Na]^+$, 906.21548 $[M+K]^+$, 1735.51034 $[2M+H]^+$, 1757.49435 $[2M+Na]^+$ (calcd. exact mass: 868.25544 $[M+H]^+$, 890.24214 $[M+Na]^+$, 906.21608 $[M+K]^+$, 1735.51312 $[2M+H]^+$, 1757.49507 $[2M+Na]^+$, formula: $C_{42}H_{49}N_3O_{11}S_3$).

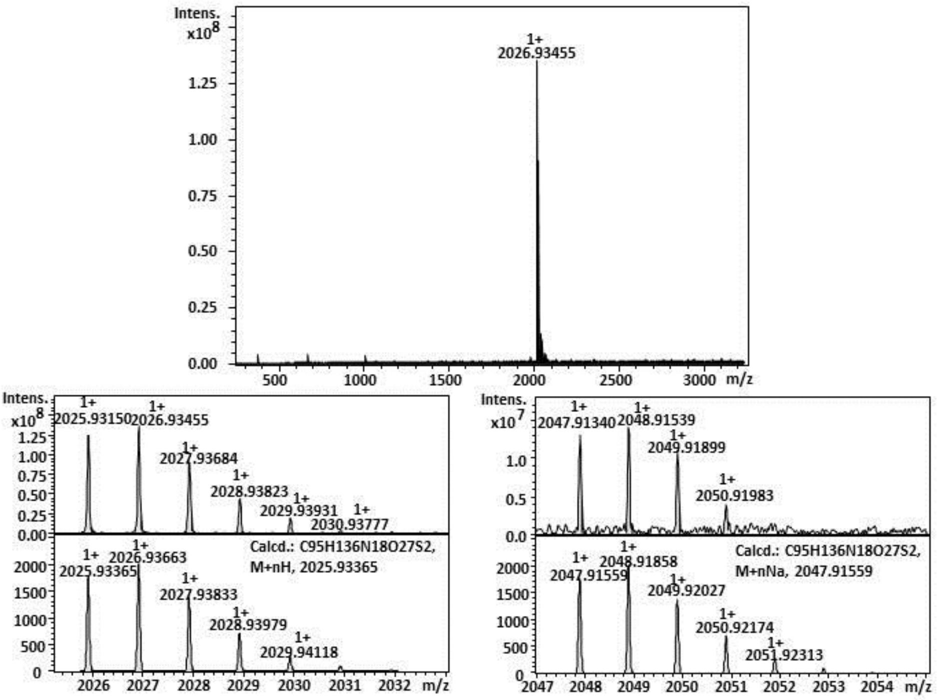

F

ig. S23 HR MALDI-FTICR-MS spectrum of ethinyl somatostatin (**7a**)**:** m/z = 2025.93150 [M+H]$^+$, 2047.91340 [M+Na]$^+$ (calcd. exact mass: 2025.93365 [M+H]$^+$, 2047.91559 [M+Na]$^+$, formula: $C_{95}H_{136}N_{18}O_{27}S_2$).

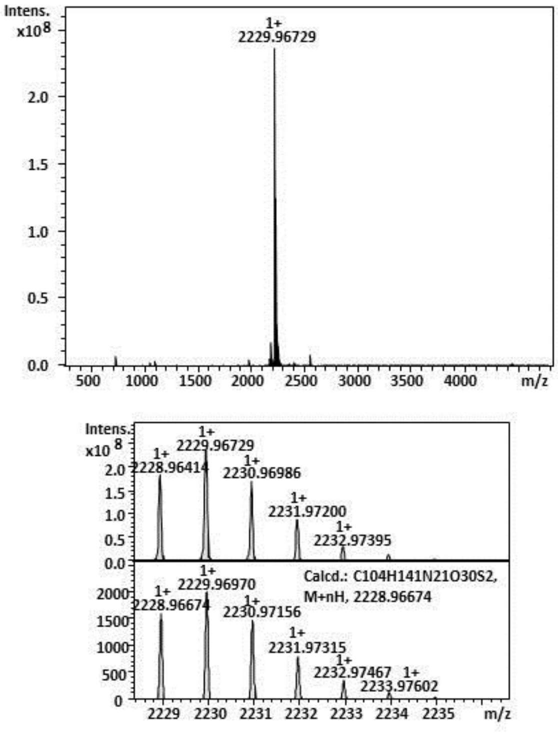

Fig. S24 HR MALDI-FTICR-MS spectrum of coumarin- somatostatin: m/z = 2228.96414 [M+H]$^+$ (calcd. exact mass: 2228.96674 [M+H]$^+$, formula: $C_{104}H_{141}N_{21}O_{30}S_2$).

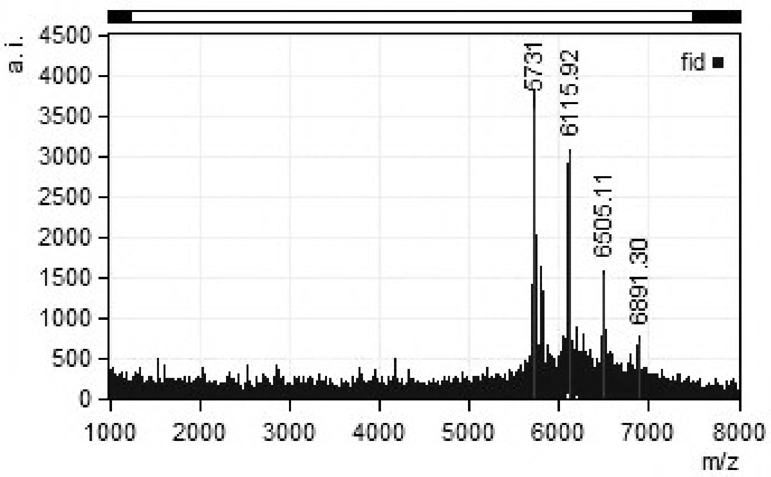

Fig. S25 MALDI-TOF-MS spectrum of multi-functionalized insulin: m/z = 5731.7 $[M+H]^+$ (calcd. exact mass: 5730.6 $[M+H]^+$, formula: $C_{254}H_{377}N_{65}O_{75}S_6$); m/z = 6115.9 $[M+H]^+$ (calcd. exact mass: 6118.8 $[M+H]^+$, formula: $C_{273}H_{409}N_{65}O_{83}S_6$); m/z = 6505.1 $[M+H]^+$ (calcd. exact mass: 6507.0 $[M+H]^+$, formula: $C_{292}H_{441}N_{65}O_{91}S_6$), m/z = 6891.3 $[M+H]^+$ (calcd. exact mass: 6895.2 $[M+H]^+$, formula: $C_{311}H_{473}N_{65}O_{99}S_6$).

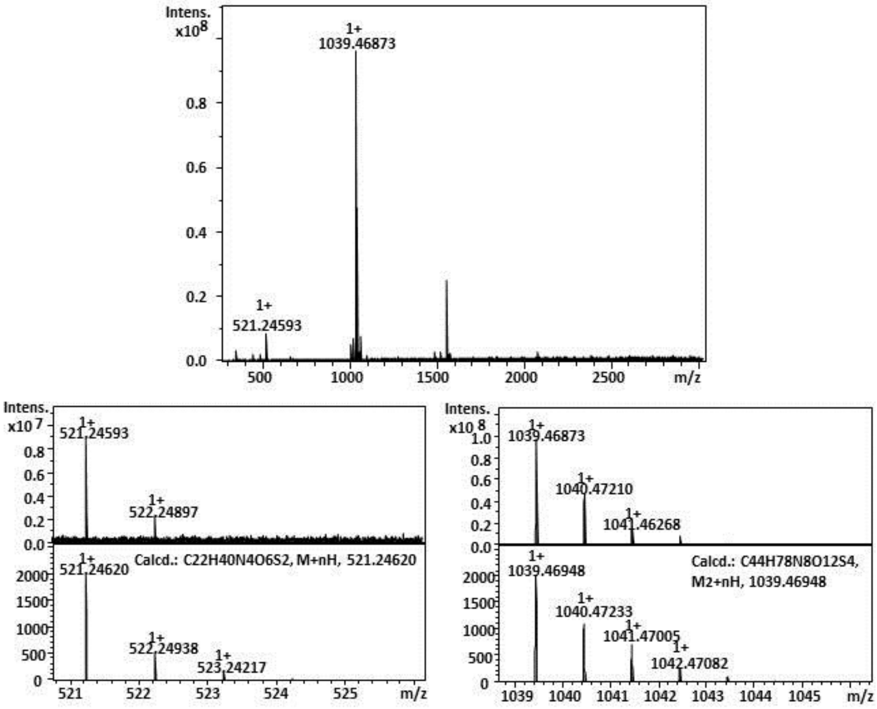

Fig. S26 HR MALDI-FTICR-MS spectrum of *N*-(1-mercapto-2-oxo-7,10,13-trioxa- 3-azahexadecan-16-yl)- 5-((3a*S*,4*S*,6a*R*)-2-oxohexahydro-1*H*-thieno[3,4-*d*]imidazol-4-yl)pentanamide (**27**): m/z = 521.24593 $[M+H]^+$, 1039.46873 $[M_2+H]^+$ (calcd. exact mass: 521.24620 $[M+H]^+$, 1039.46948 $[M_2+H]^+$, formula: $C_{22}H_{40}N_4O_6S_2$).

References

(1) Sivakumar, K.; Xie, F.; Cash, B. M.; Long, S.; Barnhill, H. N.; Wang, Q. *Org. Lett.* **2004**, *6*, 4603.

(2) Dhar, S.; Liu, Z.; Thomale, J.; Dai, H.; Lippard, S. J. *J. Am. Chem. Soc.* **2008**, *130*, 11467.

(3) Pesce, D.; Wu, Y.; Kolbe, A.; Weil, T.; Herrmann, A. *Biomaterials* **2013**, *34*, 4360.

(4) McRae, S. R.; Brown, C. L.; Bushell, G. R. *Protein Expres. Purif.* **2005**, *41*, 121.

(5) Chen, X.; Muthoosamy, K.; Pfisterer, A.;Neumann B.; Weil, T; G. R. *Bioconjugate Chem.* **2012**, *23*, 500.

(6) Wang, T.; Wu, Y.; Kuan, S. L.; Dumele, O.; Lamla, M.; Ng, D. Y. W.; Arzt, M.; Thomas J.; Mueller J., Barner-Kowollik C.; Weil T., *Chem. Eur. J.*, **2015**, *21*, 228.
